# Supplementary material for: Periodic structural changes in Pd nanoparticles during oscillatory CO oxidation reaction
Source: Nat Commun. 2022 Oct 19;13:6176. doi: 10.1038/s41467-022-33304-x (PMC9582216; doi:10.1038/s41467-022-33304-x)
Supplement: Supplementary file 1 — Supplementary Information [file 41467_2022_33304_MOESM1_ESM.pdf]

Supplementary Information for

Periodic structural changes in Pd nanoparticles during oscillatory CO oxidation reaction

Tanmay Ghosh[1, 2], Juan Manuel Arce-Ramos[3], Wen-Qing Li[3], Hongwei Yan[2], See Wee Chee[1, 2], Alexander Genest[3, 4], and Utkur Mirsaidov[1, 2, 5, 6*]

1. Department of Physics, National University of Singapore, Singapore 117551, Singapore
2. Centre for BioImaging Sciences, Department of Biological Sciences, National University of Singapore, Singapore 117557, Singapore
3. Institute of High Performance Computing, Agency for Science, Technology and Research, Singapore 138632, Singapore
4. Institute of Materials Chemistry, Technische Universität Wien, Getreidemarkt 9/BC, A-1060 Vienna, Austria
5. Centre for Advanced 2D Materials and Graphene Research Centre, National University of Singapore, Singapore 117546, Singapore
6. Department of Materials Science and Engineering, National University of Singapore, Singapore 117575, Singapore

* Corresponding author: mirsaidov@nus.edu.sg

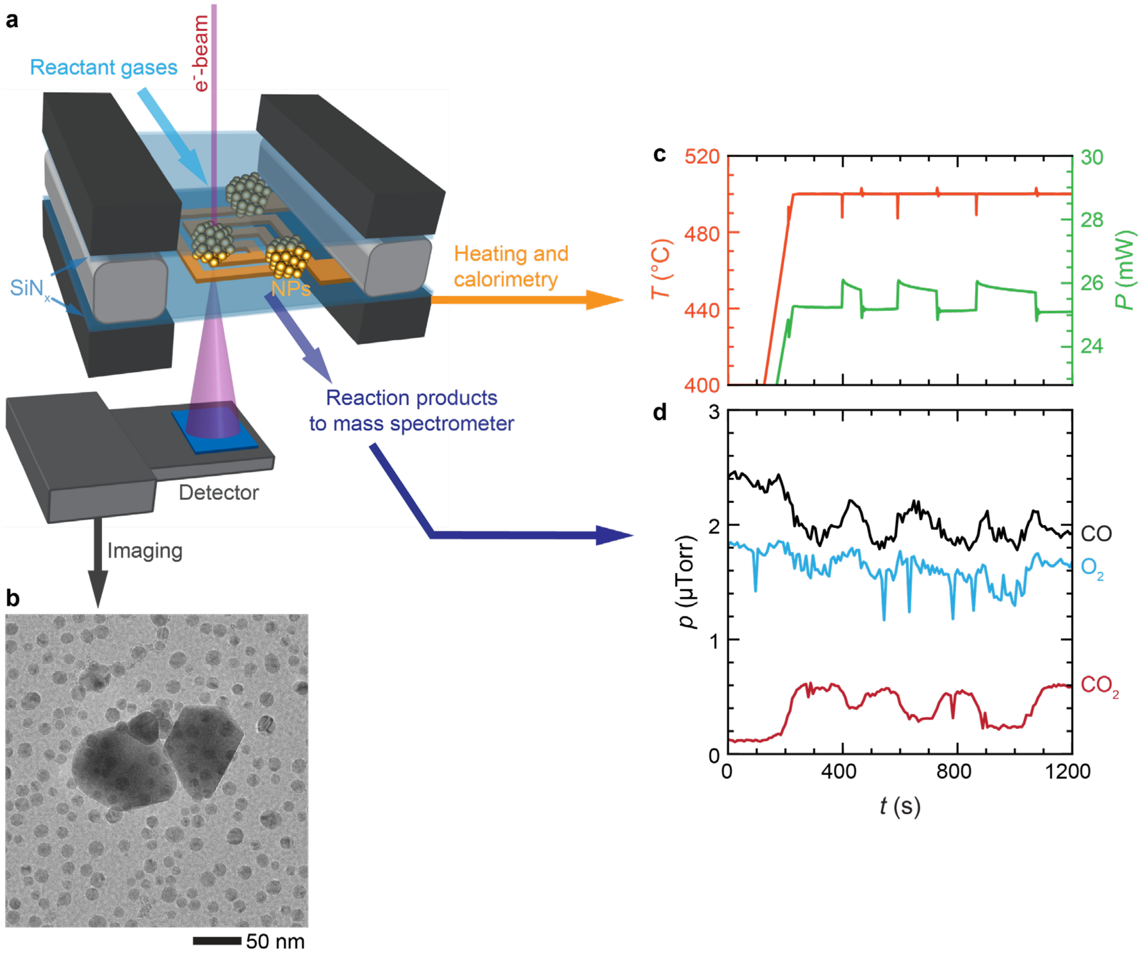

Supplementary Figure 1. Experimental setup for exploring structure–property relationship in Pd nanoparticles (NPs) during a CO oxidation reaction. (**a**) Schematic of the experimental setup where Pd NPs are encapsulated within a microfabricated gas cell with an integrated thin-film heater. (**b–c**) The NPs are imaged in a TEM under 760 Torr of 18% CO, 14% O_2, and 68% He, while the temperature (*orange curve*) and power (*green curve*) delivered to the microfabricated heater are monitored and controlled. (**d**) CO (*black curve*) and O_2 (*blue curve*) reactant, and CO_2 (*red curve*) product gases are simultaneously analyzed using an inline mass spectrometer. The setup enables three simultaneous measurements: TEM imaging of structural changes of the catalysts, monitoring the local temperature and compensated heater power, and assessing the catalytic efficiency by analyzing the reactants and products. Here, repeated fluctuations were seen in both (**c**) the heater power and (**d**) the corresponding changes in the partial pressures of CO, O_2, and CO_2 gases, indicating the oscillatory nature of the reaction.

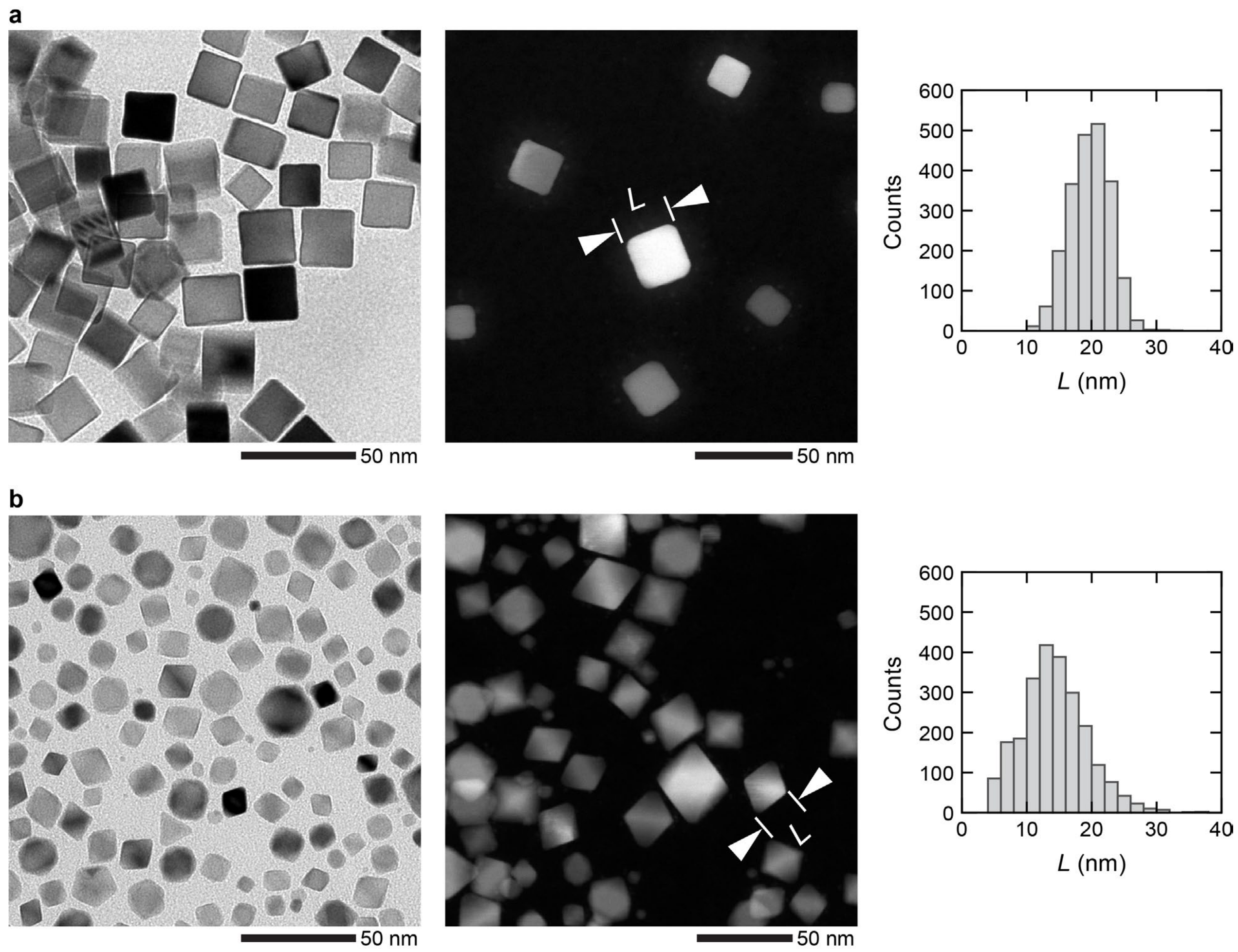

Supplementary Figure 2. Synthesized Pd NPs. TEM and STEM images and size distributions of as-synthesized (**a**) nanocubes and (**b**) nano-octahedrons.

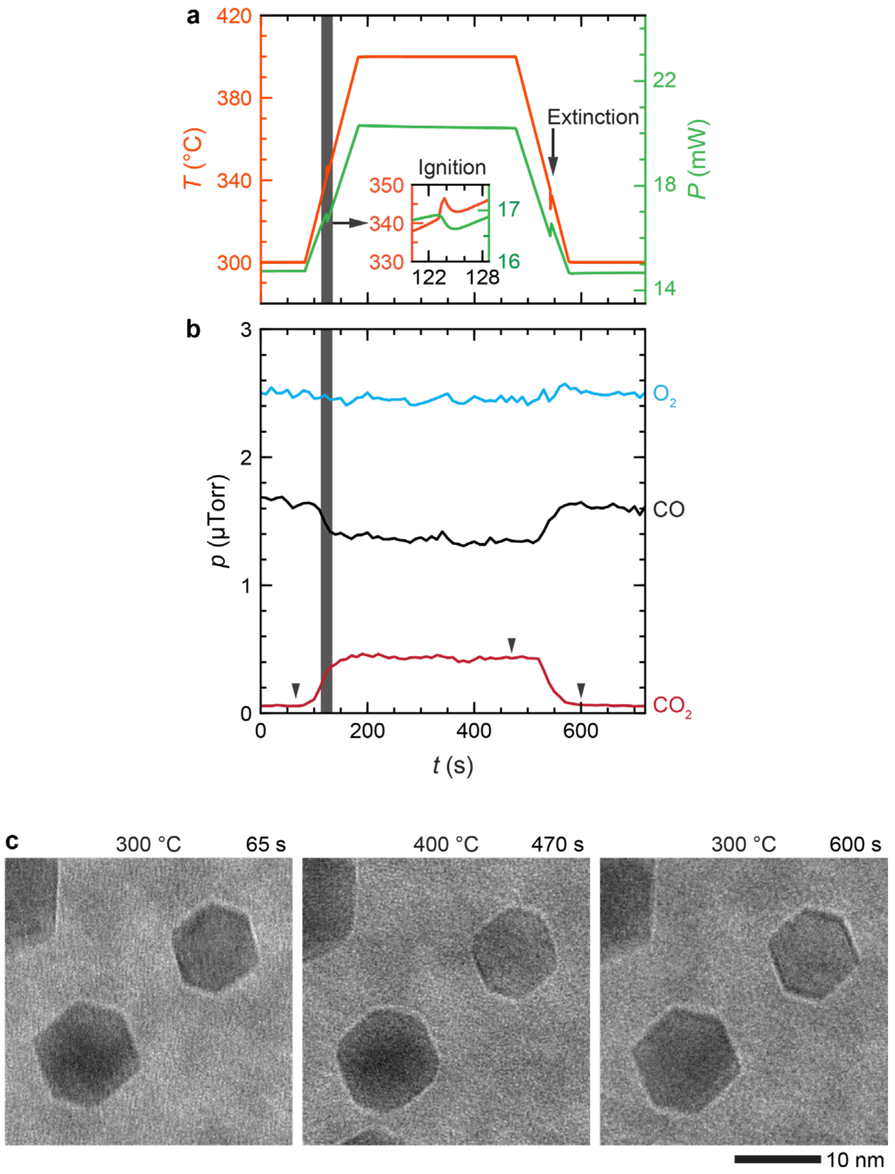

Supplementary Figure 3. Restructuring of Pd nano-octahedrons during heating and cooling. (**a**) Plots of the measured temperature, heater power, and (**b**) the corresponding amounts of CO, O_2, and CO_2 gases as the temperature was increased from 300 to 400 °C and decreased back to 300 °C at $p_{CO}/p_{O_2} \approx 0.5$. The dark gray region highlights the ignition point of the nanocatalysts. The arrows in (**b**) correspond to the timepoints of the image series shown in (**c**). (**c**) *In situ* TEM image series showing the shape of Pd nano-octahedrons during the temperature ramp-up and ramp-down from 300 to 400 °C and 400 to 300 °C, respectively. At 300 °C, the edges between {100} and {111} facets are sharp, and the NP catalysts are inactive, whereas, at 400 °C, these edges are round, and the NPs are active. The same is true for the edge between {100} and {110} facets, which are not visible in this projected image series.

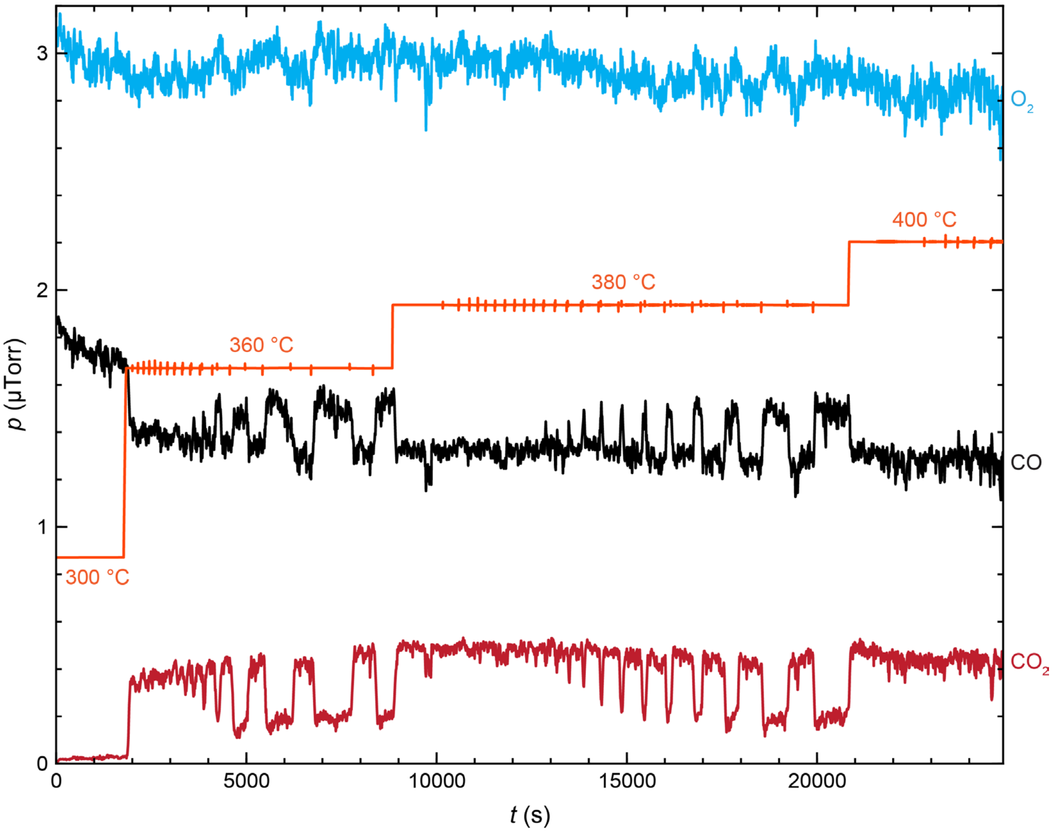

Supplementary Figure 4. Oscillatory CO oxidation reaction over Pd nano-octahedrons. A magnified version of the graph shown in Figure 1d.

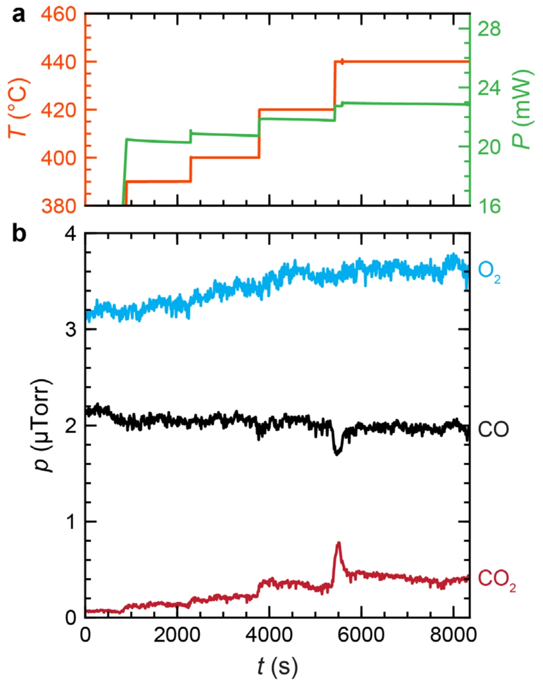

Supplementary Figure 5. Non-oscillatory CO oxidation activity over Pd nanocubes. (**a**) Plots of the measured temperature, heater power, and (**b**) the corresponding amounts of CO, O_2, and CO_2 gases during the reaction at temperatures of 390, 400, 420, and 440 °C, and at $p_{CO}/p_{O_2} \approx 0.5$.

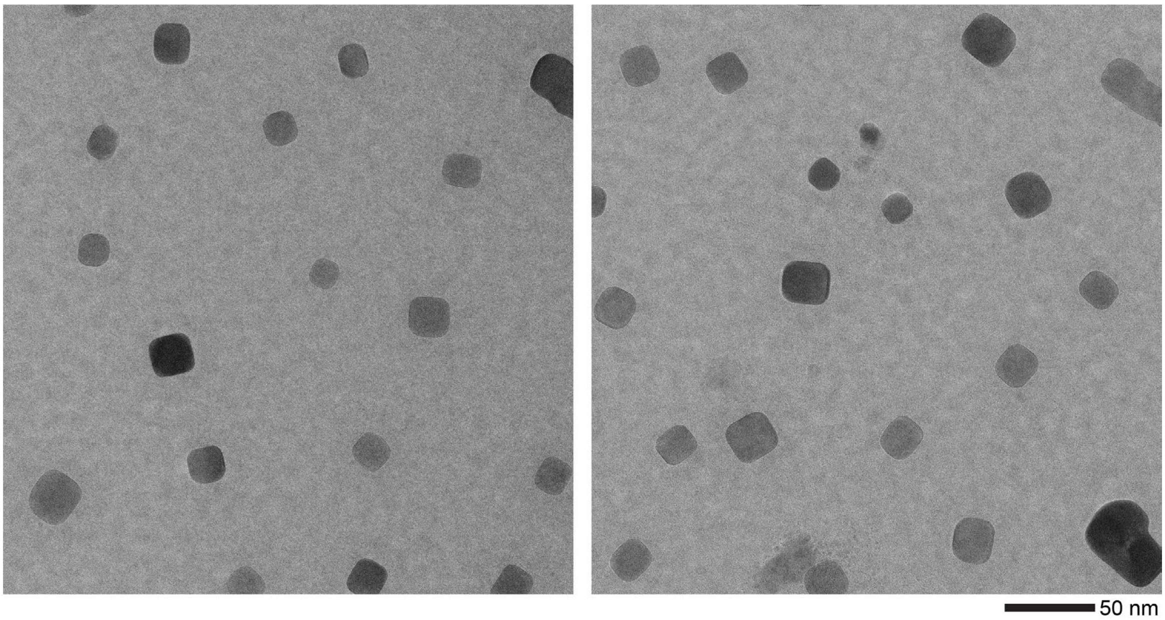

Supplementary Figure 6. Post-reaction TEM images of Pd nanocubes. Low-magnification TEM images of Pd nanocubes after a 2-hour-long *in situ* CO oxidation reaction, confirming that their cubic structure was preserved after the reaction.

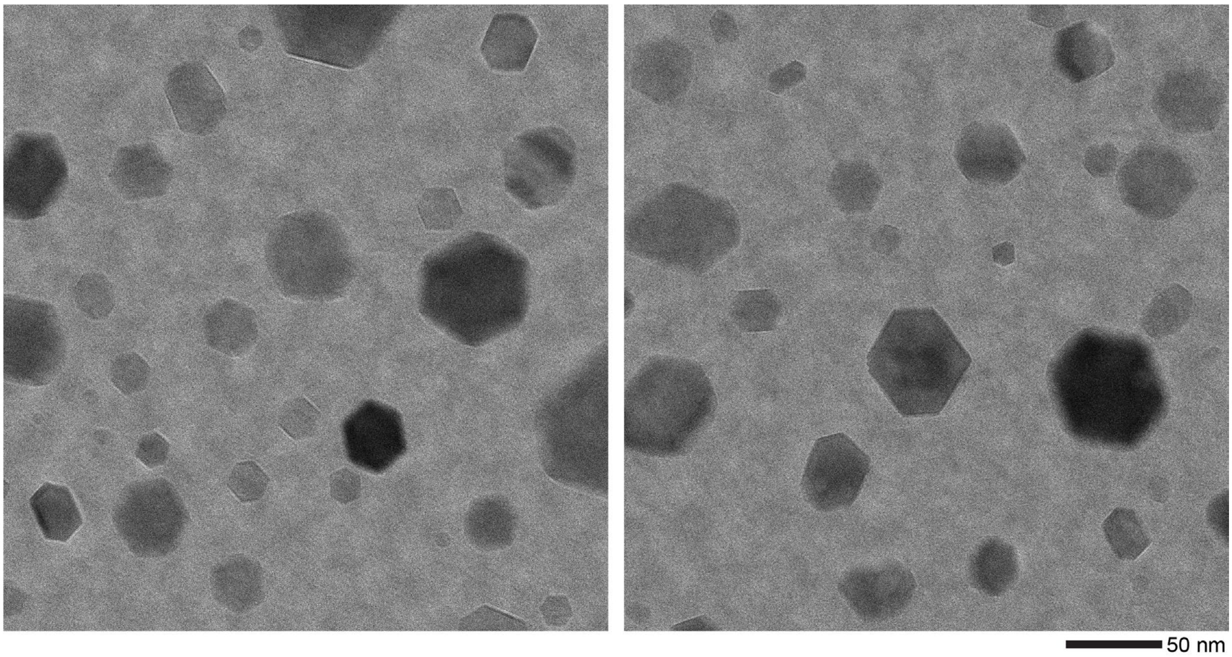

Supplementary Figure 7. Post-reaction TEM images of Pd nano-octahedrons. Low-magnification TEM images of Pd nano-octahedrons after an 8-hour-long *in situ* CO oxidation reaction showing very small changes in the shape of the NPs after the reaction (*i.e.*, NPs maintain their overall octahedral shape).

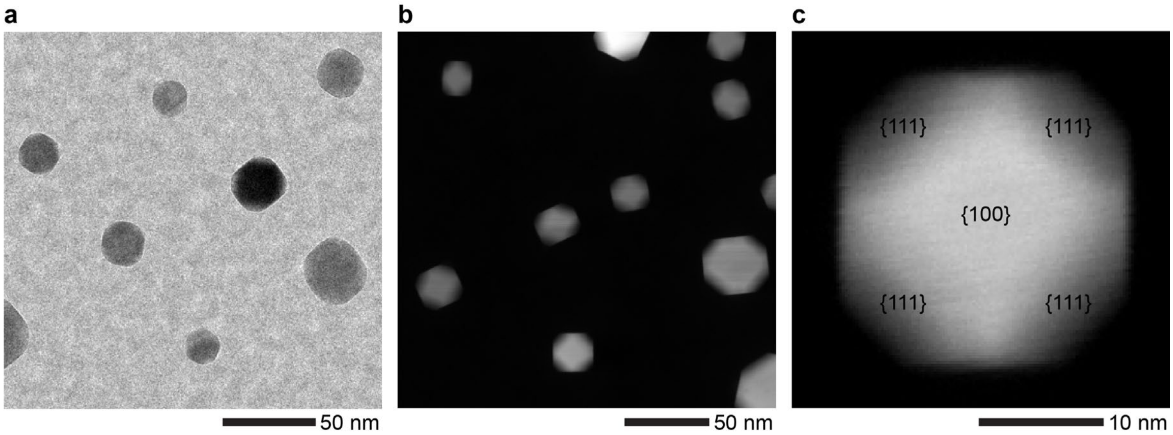

Supplementary Figure 8. Conversion of Pd nanocubes into truncated nanocubes. (**a**) TEM and (**b**) low- and (**c**) higher-magnification STEM images of truncated Pd nanocubes obtained by heating the nanocubes at 300 °C in a 20% O_2 and 80% He environment inside a gas cell.

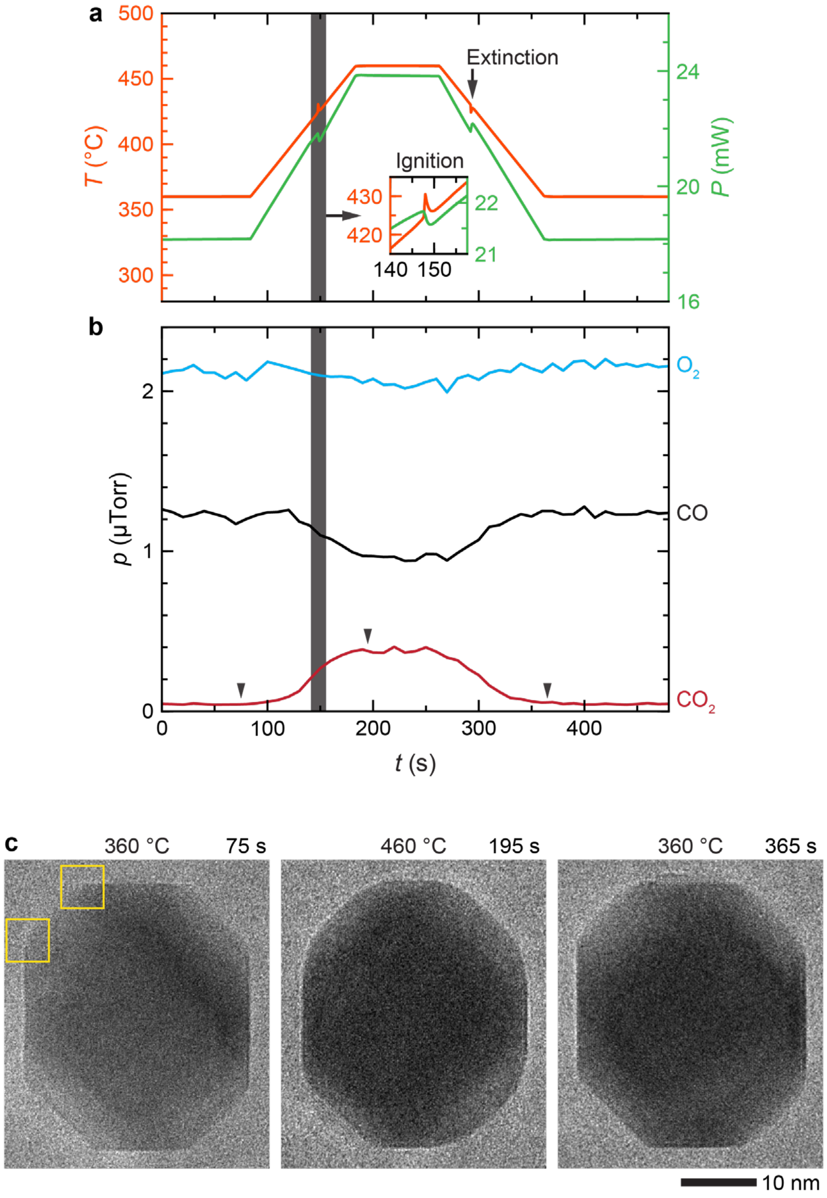

Supplementary Figure 9. Restructuring of a truncated Pd nanocube during heating and cooling. (**a**) Plots of the measured temperature, heater power, and (**b**) the corresponding amounts of CO, O_2, and CO_2 gases as the temperature was increased from 360 to 460 °C and decreased back to 360 °C at $p_{CO}/p_{O_2} \approx 0.5$. The arrows in (**b**) correspond to the timepoints of the image series shown in (**c**). (**c**) *In situ* TEM image series showing the shape of a truncated Pd nanocube during the temperature ramp-up and ramp-down from 360 to 460 °C and 460 to 360 °C, respectively. At 360 °C, the edges between {100} and {110} facets (*yellow boxes*) are sharp, and the NP catalyst is inactive, whereas, at 460 °C, these edges are round, and the NP is active. The same is true for the edge between {100} and {111} facets, which are not visible in this projected image series.

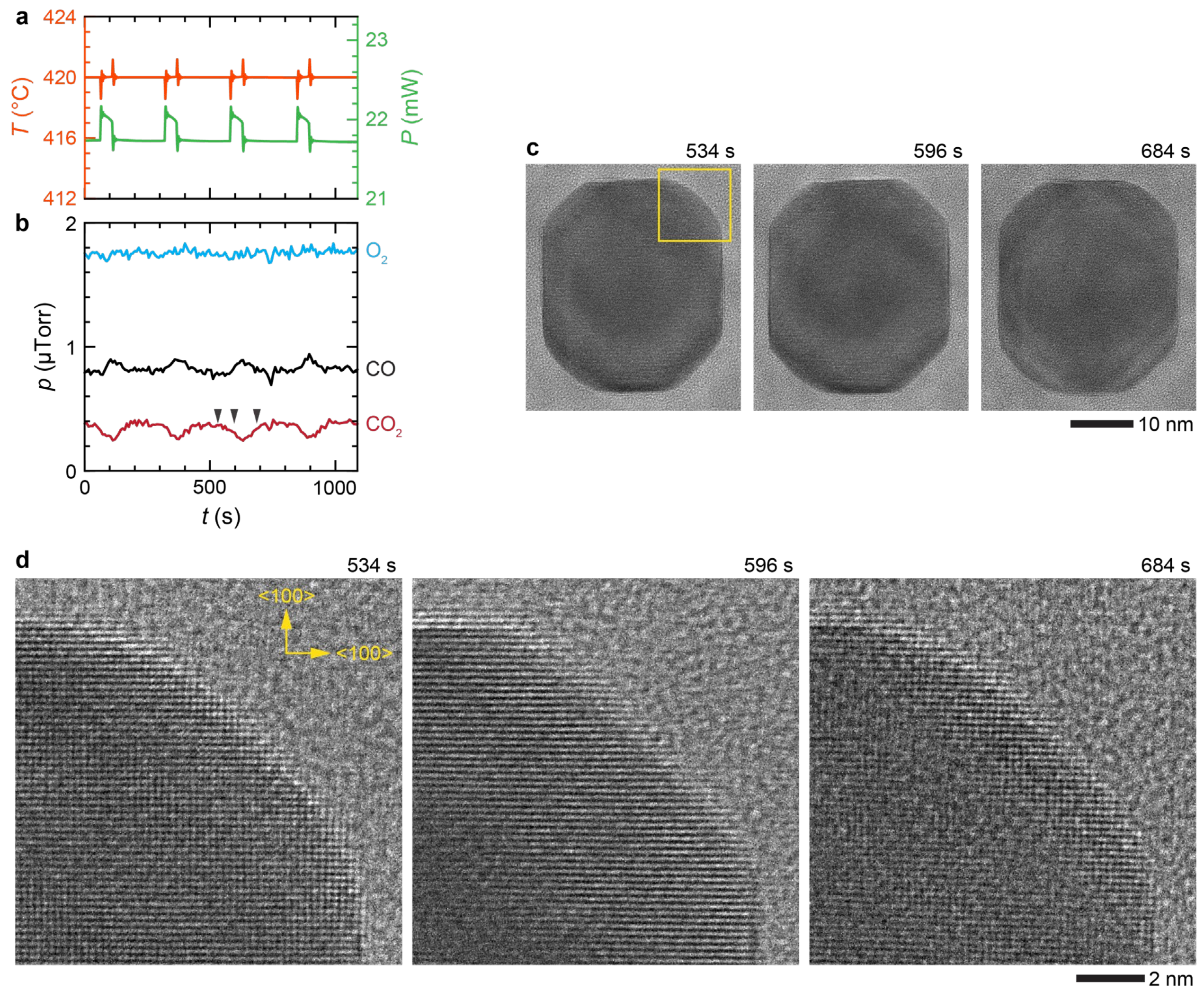

Supplementary Figure 10. Restructuring of the truncated Pd nanocube shown in Figure 3 during 500 s < *t* < 700 s. (**a**) Plots of the measured temperature, heater power, and (**b**) the corresponding amounts of CO, O_2, and CO_2 gases during the oscillatory CO oxidation reaction at 420 °C and $p_{CO}/p_{O_2} \approx 0.5$. The arrows in (**b**) correspond to the timepoints of the image series shown in (**c–d**). (**c**) *In situ* TEM image series of a truncated nanocube in low- and high-activity states during the oscillation. (**d**) High-resolution image series of the NP's corner, as indicated by the yellow box in (**c**). During the reaction, the corners of the truncated nanocube restructure between round (at 534 and 684 s) and flat (at 596 s) facets (Supplementary Movie 2).

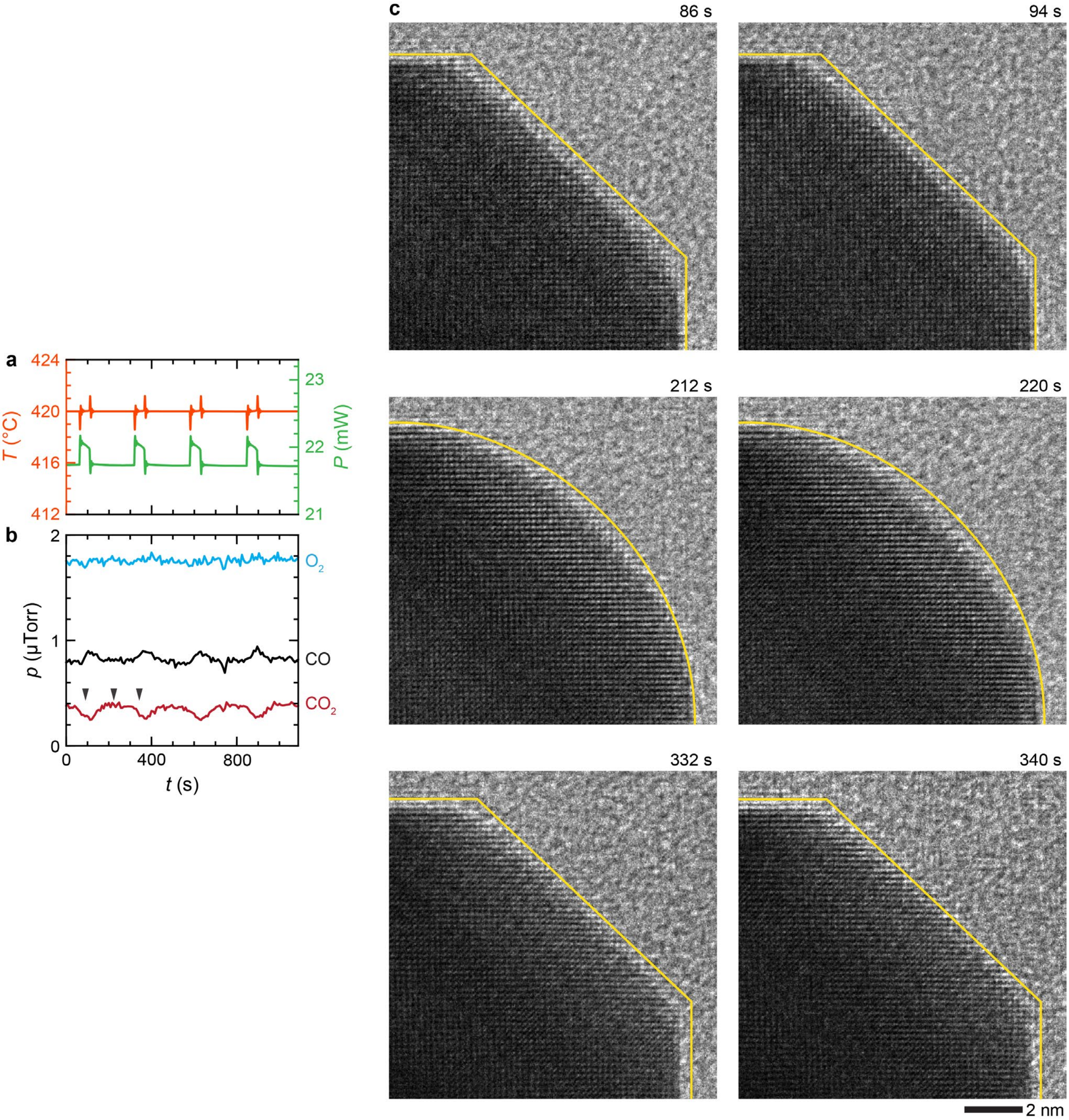

Supplementary Figure 11. Restructuring of the truncated Pd nanocube shown in Figure 3. (**a**) Plots of the measured temperature, heater power, and (**b**) the corresponding amounts of CO, O_2, and CO_2 gases during the oscillatory CO oxidation reaction 420 °C and $p_{CO}/p_{O_2} \approx 0.5$. The arrows in (**b**) correspond to the timepoints of the image series shown in (**c**). (**c**) High-resolution TEM image series of the truncated nanocube shown in Figure 3d at 86, 94, 212, 220, 332, and 340 s. During the reaction, the corners of the truncated nanocube restructure between round (at 212 and 220 s) and flat (at 86, 94, 332, and 340 s) facets (Supplementary Movie 2).

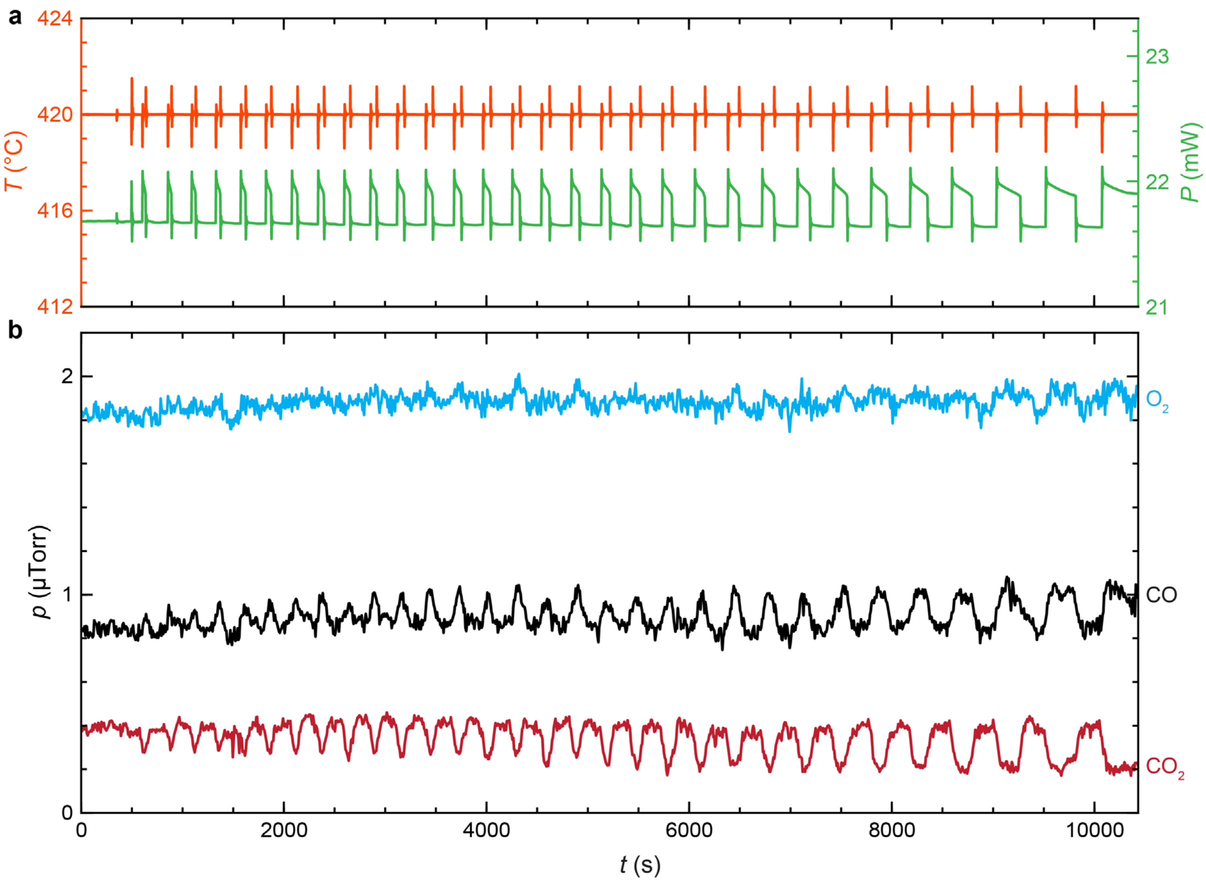

Supplementary Figure 12. Extended oscillatory CO oxidation reaction over truncated Pd nanocubes. (**a**) Plots of the measured temperature, heater power, and (**b**) the corresponding amounts of CO, O_2, and CO_2 gases during the reaction at 420 °C and $p_{CO}/p_{O_2} \approx 0.5$.

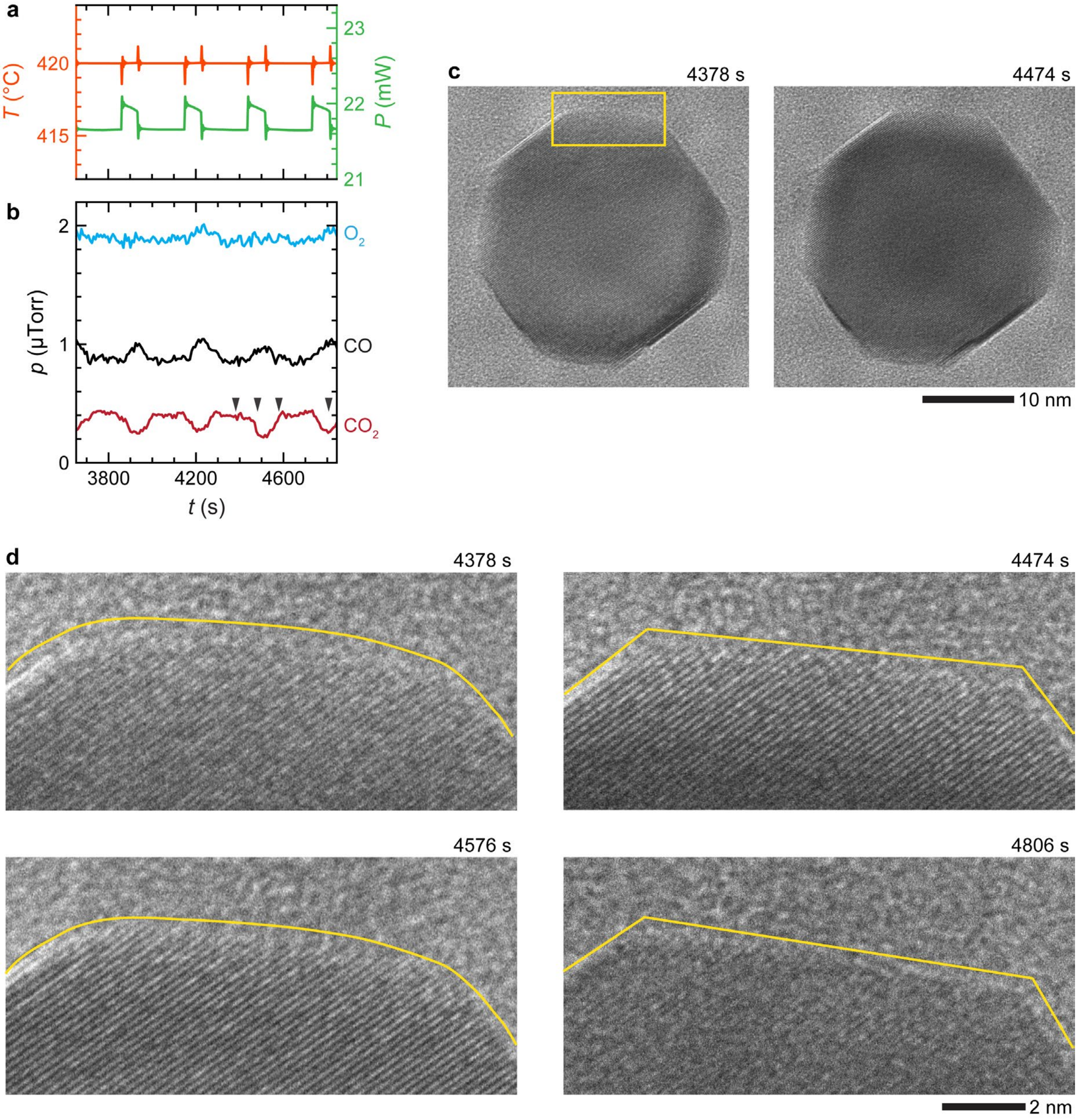

Supplementary Figure 13. Restructuring of a truncated Pd nanocube during 3650 s < *t* < 4850 s as shown in Supplementary Figure 12. (**a**) Plots of the measured temperature, heater power, and (**b**) the corresponding amounts of CO, O_2, and CO_2 gases during the oscillatory CO oxidation reaction at 420 °C and $p_{CO}/p_{O_2} \approx 0.5$. The arrows in (**b**) correspond to the timepoints of the image series shown in (**c–d**). (**c**) *In situ* TEM images of a truncated nanocube in low- and high-activity states during the oscillation. (**d**) High-resolution image series of the NP's corner, as indicated by the yellow box in (**c**). During the reaction, the corners of the truncated nanocube restructure between round (at 4378 and 4576 s) and flat (at 4474 and 4806 s) facets.

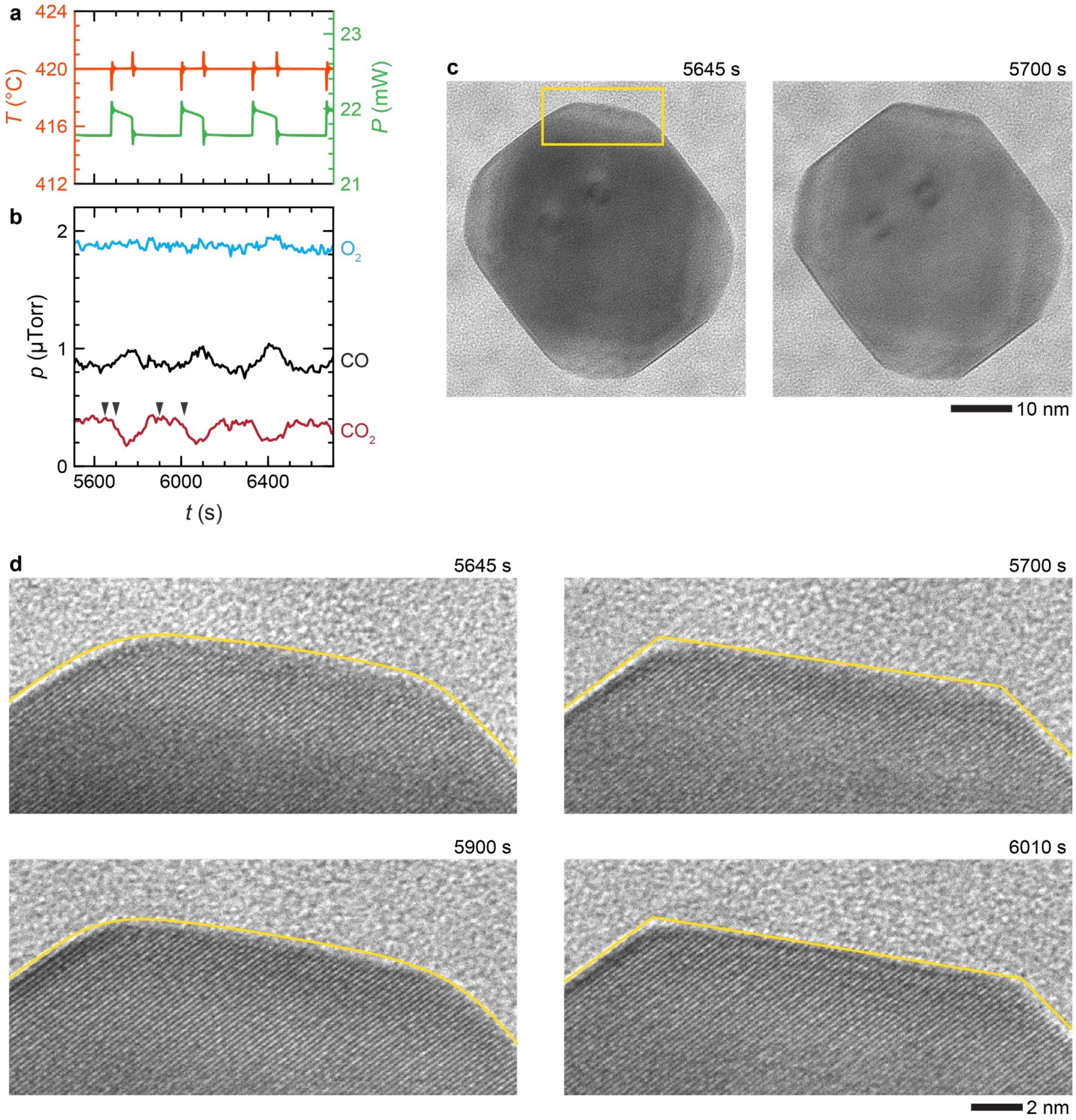

Supplementary Figure 14. Restructuring of a truncated Pd nanocube during 5500 s < *t* < 6700 s as shown in Supplementary Figure 12. (**a**) Plots of the measured temperature, heater power, and (**b**) the corresponding amounts of CO, O_2, and CO_2 gases during the oscillatory CO oxidation reaction at 420 °C and $p_{CO}/p_{O_2} \approx 0.5$. The arrows in (**b**) correspond to the timepoints of the image series shown in (**c–d**). (**c**) *In situ* TEM image series of a truncated nanocube in low- and high-activity states during the oscillation. (**d**) High-resolution image series of the NP's corner, as indicated by the yellow box in (**c**). During the reaction, the corners of the truncated nanocube restructure between round (at 5645 and 5900 s) and flat (at 5700 and 6010 s) facets.

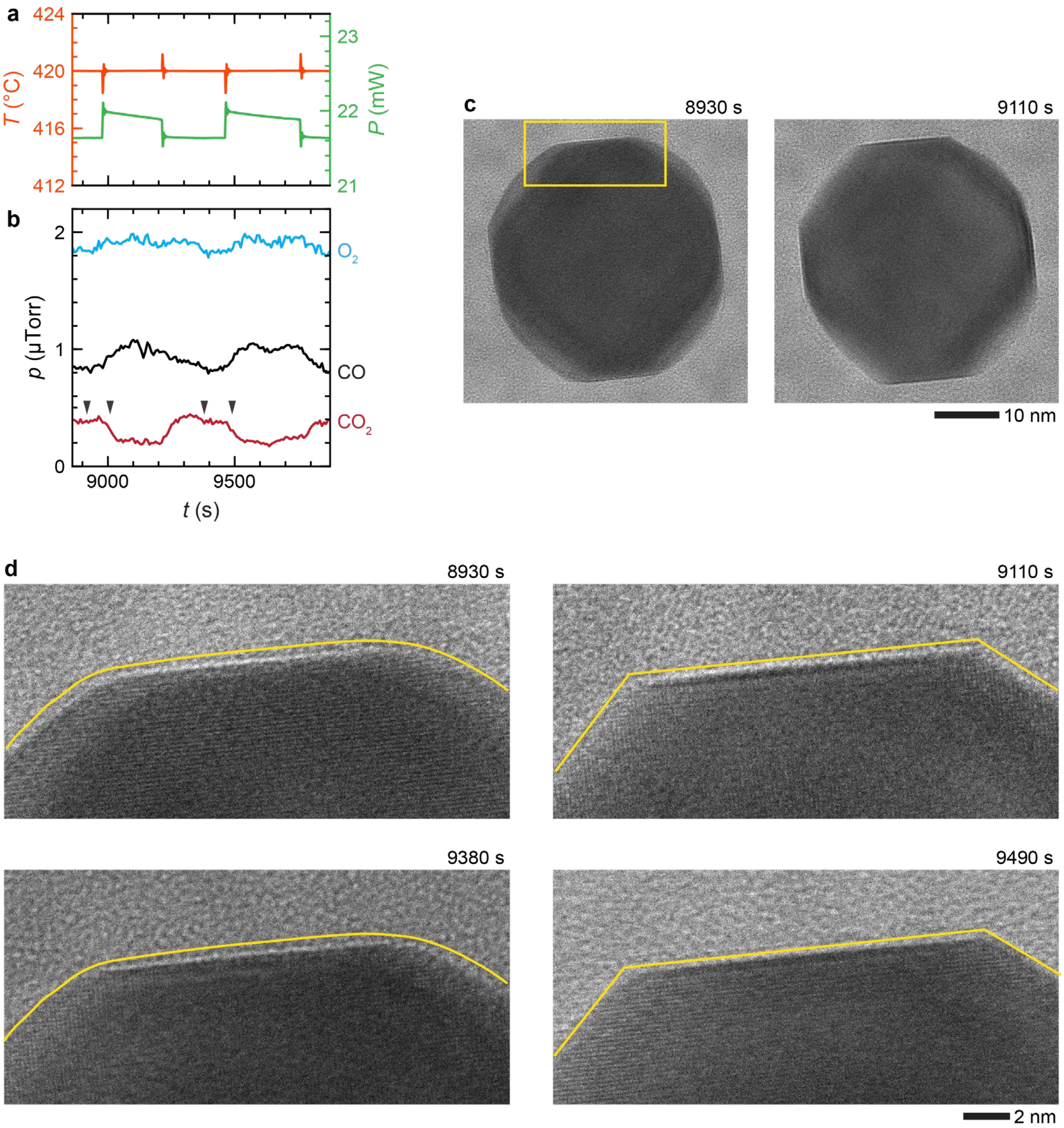

Supplementary Figure 15. Restructuring of a truncated Pd nanocube during 8875 s < *t* < 9875 s as shown in Supplementary Figure 12. (**a**) Plots of the measured temperature, heater power, and (**b**) the corresponding amounts of CO, O_2, and CO_2 gases during the oscillatory CO oxidation reaction at 420 °C and $p_{CO}/p_{O_2} \approx 0.5$. The arrows in (**b**) correspond to the timepoints of the image series shown in (**c–d**). (**c**) *In situ* TEM image series of a truncated nanocube in low- and high-activity states during the oscillation. (**d**) High-resolution image series of the NP's edge, as indicated by the yellow box in (**c**). During the reaction, the corners of the truncated nanocube restructure between round (at 8930 and 9380 s) and flat (at 9110 and 9490 s) facets.

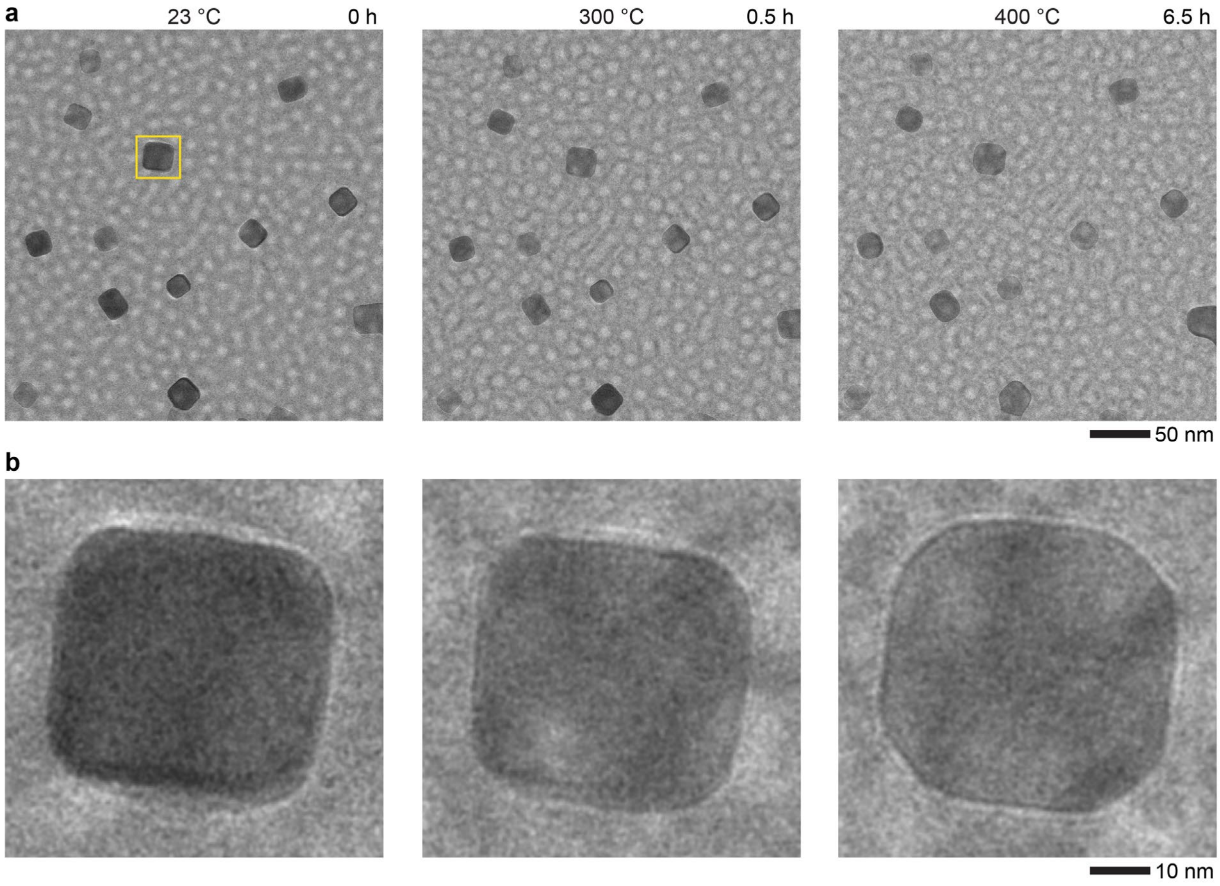

Supplementary Figure 16. Transformation of Pd nanocubes to truncated nanocubes during extended heating. (**a**) *In situ* TEM image series of Pd nanocubes at $p_{\mathrm{CO}}/p_{\mathrm{O_2}} \approx 0.5$ and at different temperatures showing their transformation into truncated nanocubes after 6 h of heating at 400 °C. (**b**) Higher-magnification TEM images of a Pd nanocube cropped from an area indicated by the yellow box in (**a**), highlighting the truncation. These truncated nanocubes display oscillatory reactivity, as shown in Supplementary Figure 17.

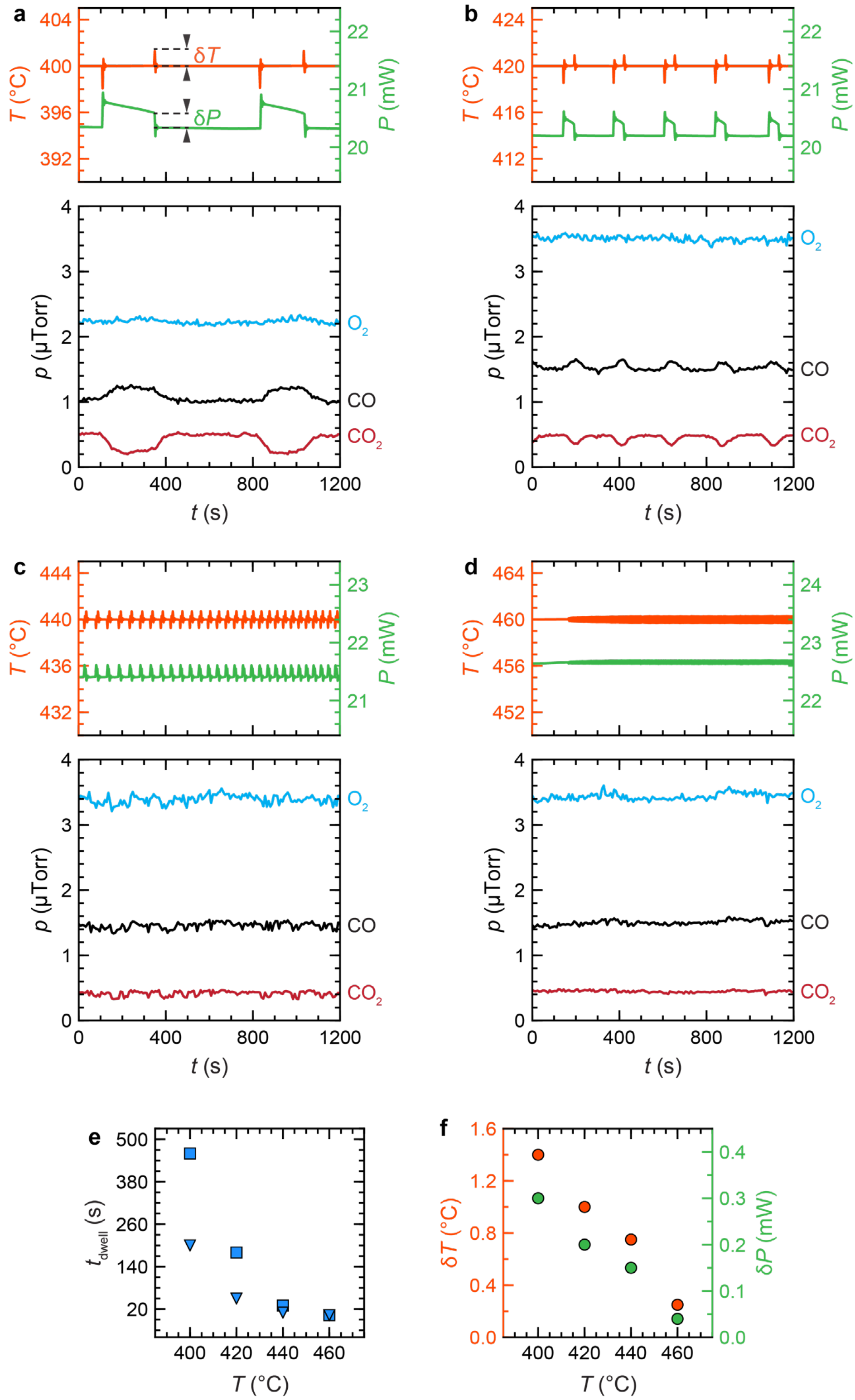

Supplementary Figure 17. Comparison of oscillatory patterns from truncated Pd nanocubes at different reaction temperatures. Plots of the measured temperatures, heater powers, and the corresponding amounts of CO, O_2, and CO_2 gases during the oscillatory CO oxidation reaction at $p_{CO}/p_{O_2} \approx 0.5$ and at temperatures of (**a**) 400 °C, (**b**) 420 °C, (**c**) 440 °C, and (**d**) 460 °C. (**e**) High- (*blue squares*) and low-activity (*blue triangles*) dwell times as a function of the reaction temperature. (**f**) The temperature jump (δT, *orange circles*) and concurrent drop in heater power (δP, *green circles*) as a function of the reaction temperature at the onset of high-activity states of the oscillation. δT and δP are defined by arrows in (**a**). Note that these truncated nanocubes are the transformed nanocubes shown in Supplementary Figure 16.

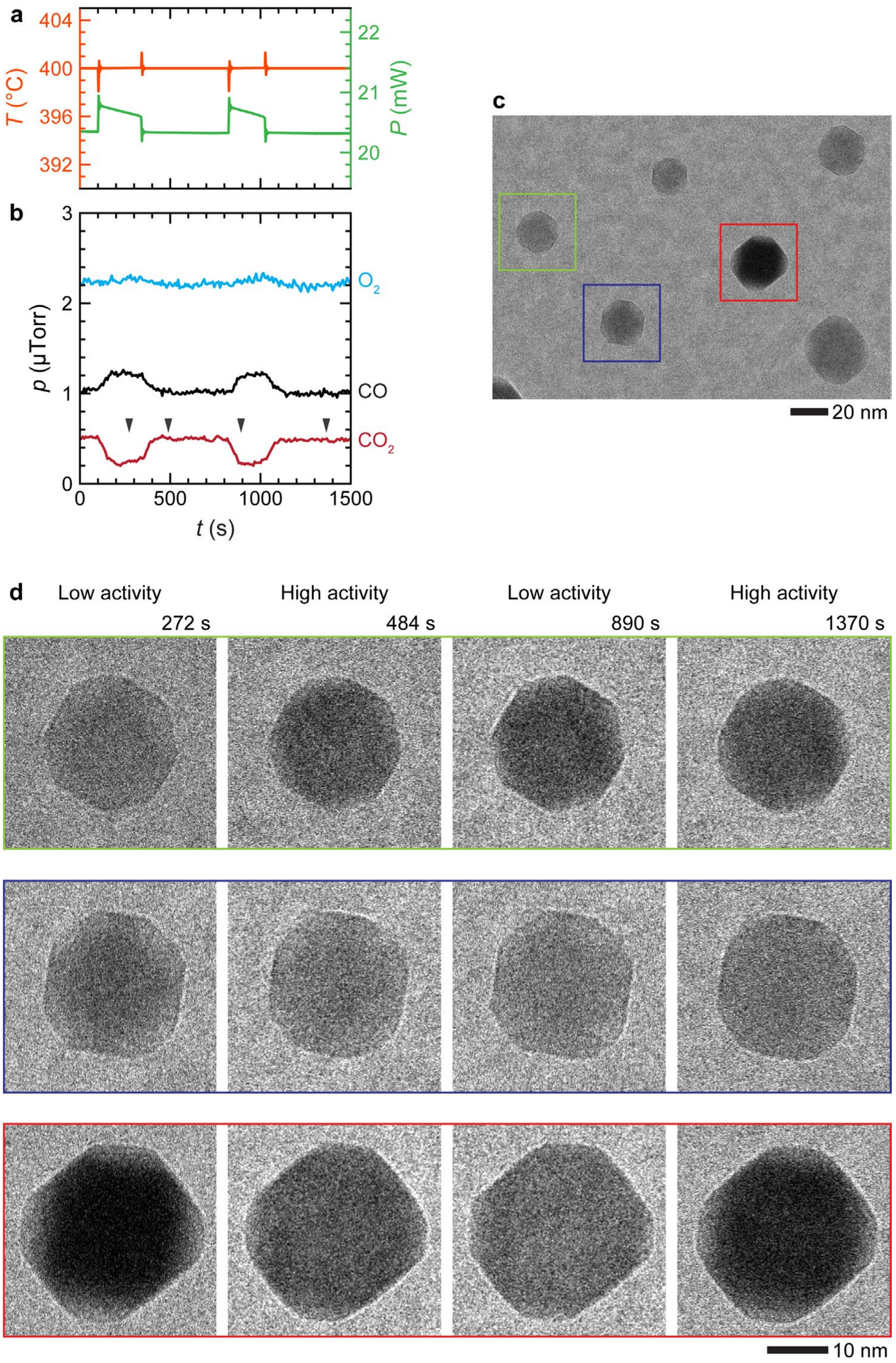

Supplementary Figure 18. TEM images of multiple truncated Pd nanocubes during an oscillatory reaction at 400 °C. (**a**) Plots of the measured temperature, heater power, and (**b**) the corresponding amounts of CO, O_2, and CO_2 gases during the oscillatory CO oxidation reaction at 400 °C and $p_{CO}/p_{O_2} \approx 0.5$. The arrows in (**b**) correspond to the timepoints of the image series shown in (**d**). (**c**) Low-magnification TEM image of six truncated Pd nanocubes during the reaction. (**d**) *In situ* TEM image series of three individual nanocubes shown in (**c**) during the oscillation. This result confirms that truncated nanocubes restructure synchronously during the reaction inside the gas cell.

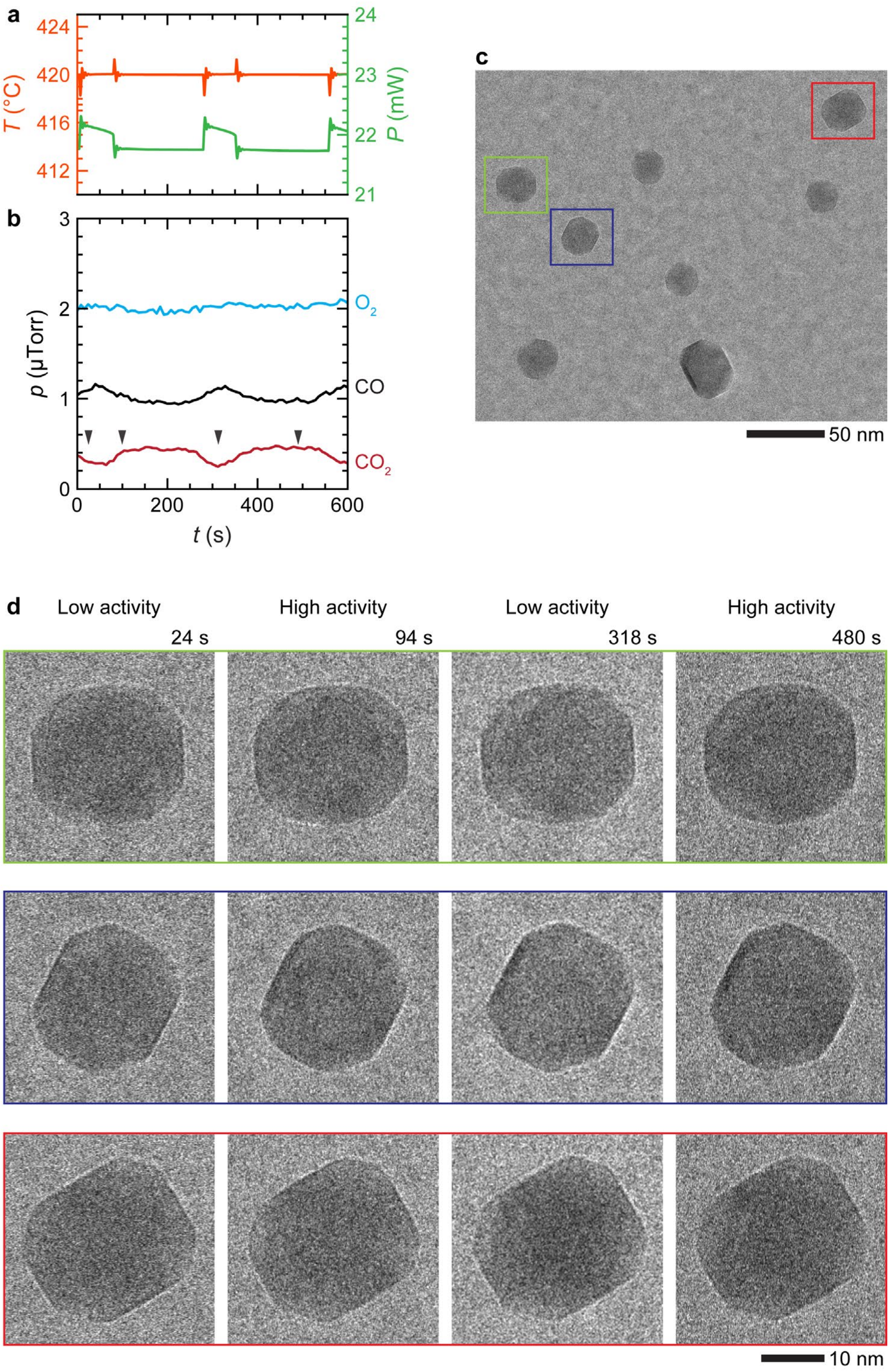

Supplementary Figure 19. TEM images of multiple truncated Pd nanocubes during an oscillatory reaction at 420 °C. (**a**) Plots of the measured temperature, heater power, and (**b**) the corresponding amounts of CO, O_2, and CO_2 gases during the oscillatory CO oxidation reaction at 420 °C and $p_{CO}/p_{O_2} \approx 0.5$. The arrows in (**b**) correspond to the timepoints of the image series shown in (**d**). (**c**) Low-magnification TEM image of eight truncated Pd nanocubes during the reaction. (**d**) *In situ* TEM image series of four individual nanocubes shown in (**c**) during the oscillation. This result confirms that truncated nanocubes synchronously restructure during the reaction inside the gas cell.

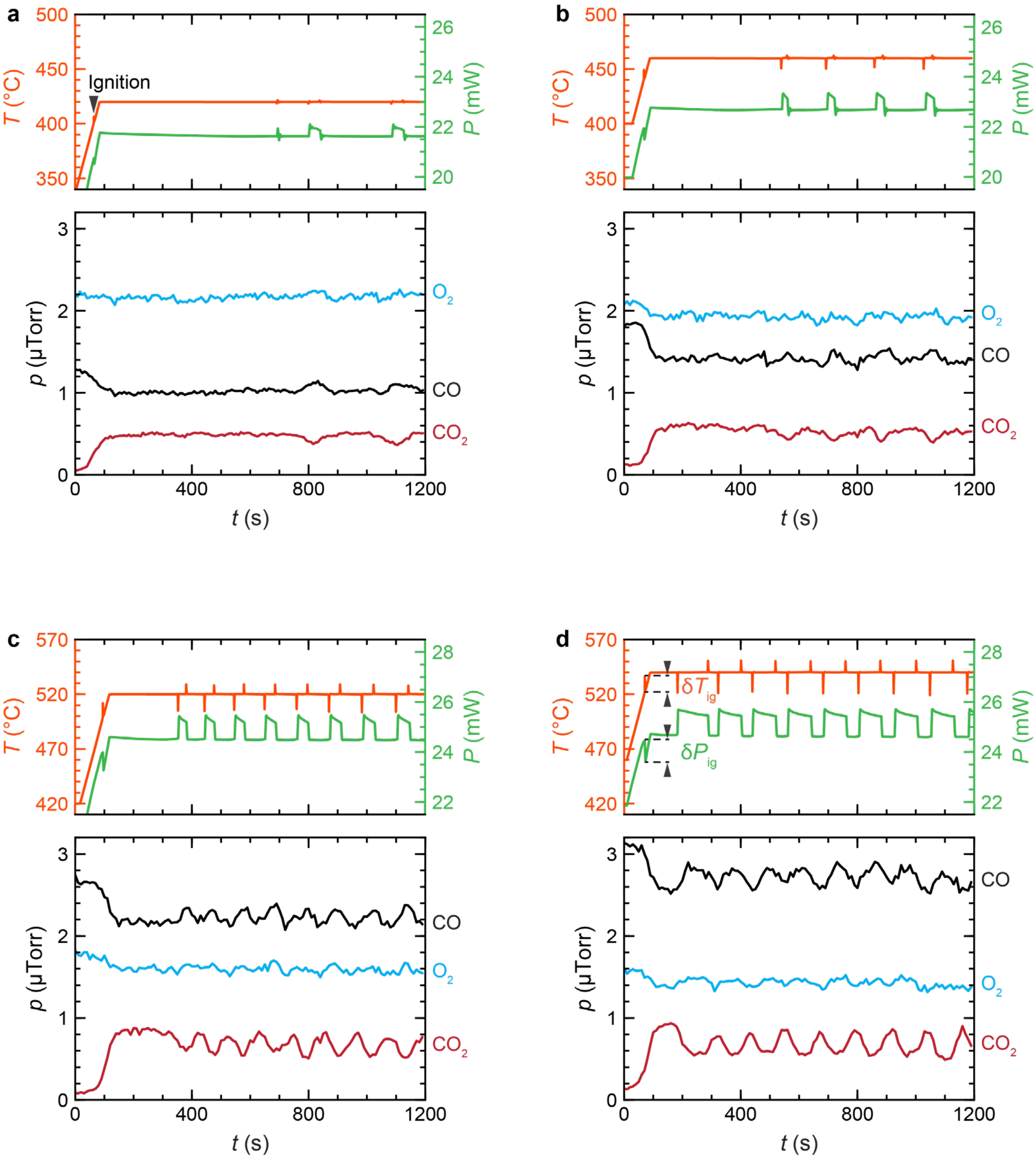

Supplementary Figure 20. Comparison of oscillatory wave patterns from truncated Pd nanocubes at different p_{CO}/p_{O_2} ratios. Plots of the measured temperatures, heater powers, and corresponding amounts of CO, O_2, and CO_2 gases during reaction at p_{CO}/p_{O_2} of (**a**) 0.5, (**b**) 0.8, (**c**) 1.5, and (**d**) 2.0. Note that the reaction temperature is different in each experiment. Since the ignition temperature increases with the increase in p_{CO}, we needed to increase the reaction temperature to see the oscillations. The ignition-induced temperature jump (δT_{ig}) and concurrent drop in the heater power (δP_{ig}) are indicated by arrows in (**d**), and their values as a function of p_{CO}/p_{O_2} are summarized in Figure 4e.

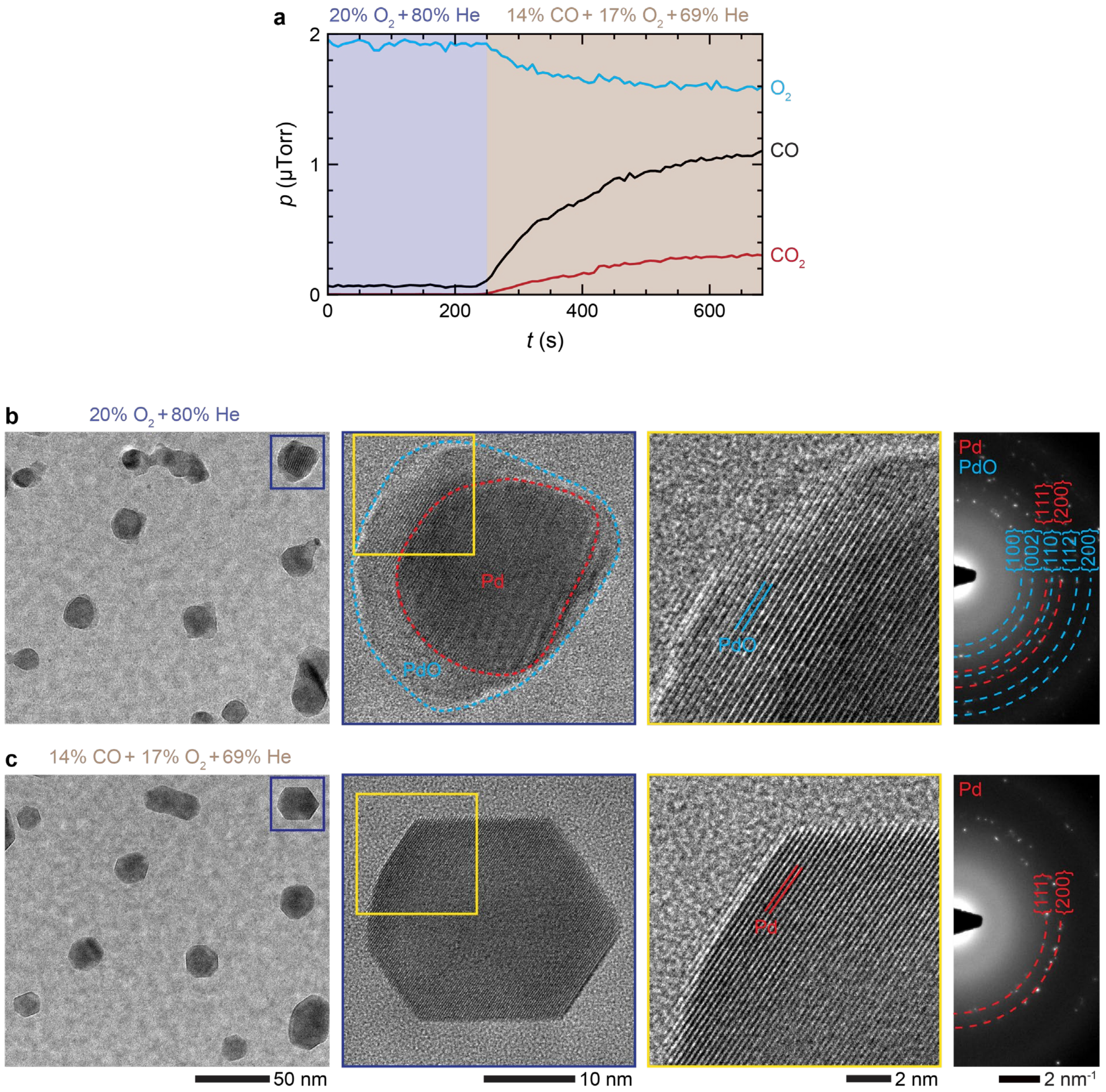

Supplementary Figure 21. Restructuring of Pd NPs under O_2 and a mixture of O_2 and CO environments. (**a**) Plots of the amounts of CO, O_2, and CO_2 gases during the CO oxidation reaction at 460 °C. (**b**) TEM and electron diffraction images of Pd NPs under 20% O_2 and 80% He environment at 460 °C showing that the surface of these NPs is oxidized. (**c**) TEM and electron diffraction images of the same Pd NPs after switching the gaseous environment to $p_{CO}/p_{O_2} \approx 0.8$ atmosphere (*i.e.*, 14% CO, 17% O_2, and 69% He) while maintaining the temperature at 460 °C. Here, the surface PdO vanishes by rapidly reducing into metallic Pd. Supplementary Figure 22 shows the oscillatory behavior of the same NPs.

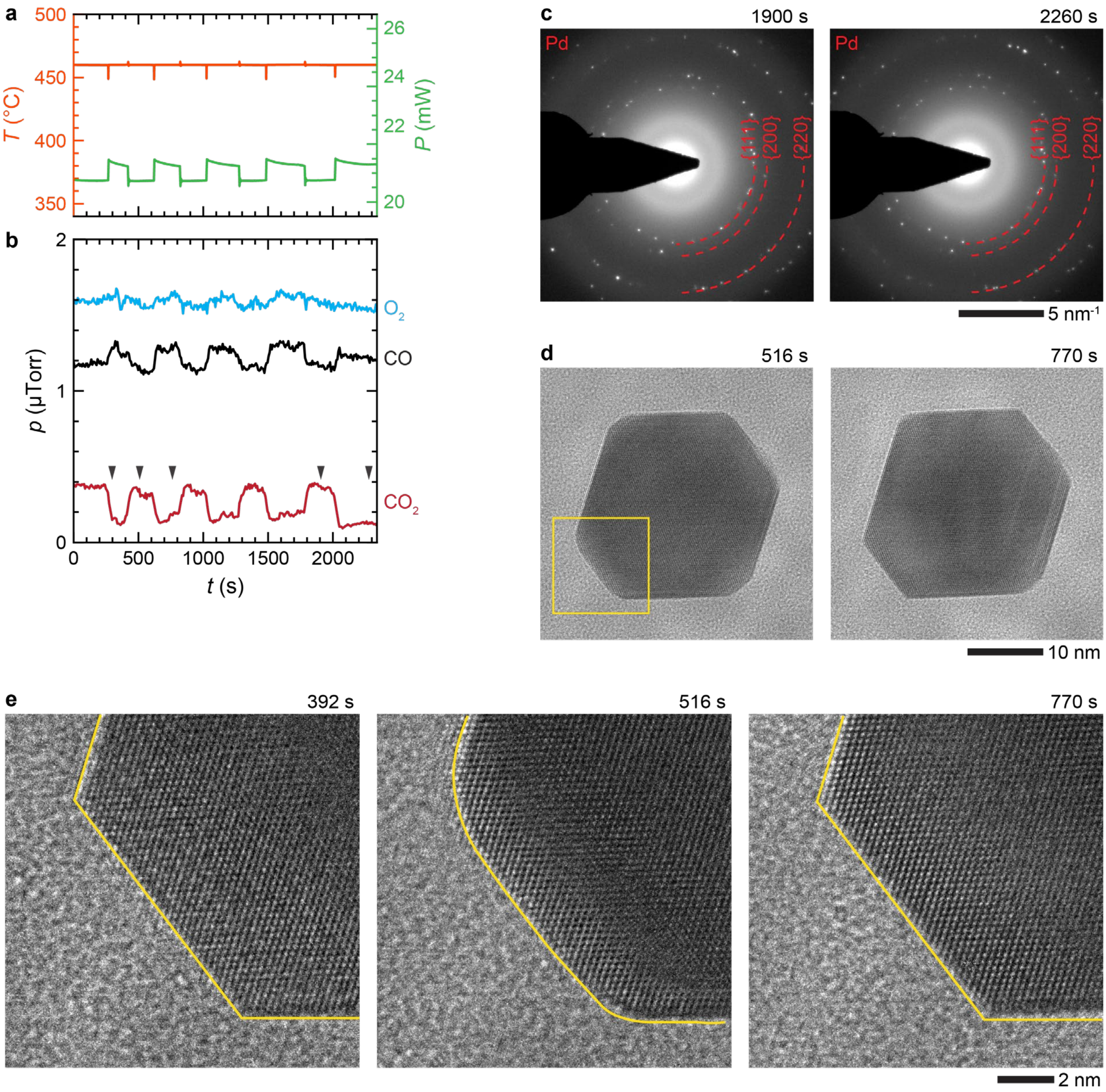

Supplementary Figure 22. Reaction oscillation from the same NPs and under the same conditions stated in Supplementary Figure 21. (**a**) Plots of the measured temperature, heater power, and (**b**) the corresponding amounts of CO, O_2, and CO_2 gases during the oscillatory CO oxidation reaction at 460 °C and $p_{CO}/p_{O_2} \approx 0.8$. The arrows in (**b**) correspond to the timepoints of the image series shown in (**c**–**e**). (**c**) *In situ* electron diffraction images taken at low- and high-activity states during the oscillation showing that Pd NPs do not have any detectable amount of PdO during the reaction. (**d**) *In situ* TEM image series of a Pd NP in low- and high-activity states during the oscillation. (**e**) High-resolution image series of the NP's corner, as indicated by the yellow box in (**d**). During the reaction, the corners of the NP restructure between round (at 516 s) and flat (at 392 and 770 s) facets.

Supplementary Note

1. DFT and thermodynamic calculations

All DFT calculations were done using the plane-wave DFT software VASP[1-4] and the projector-augmented wave method.[5] We used the BEEF-vdW functional since it can reliably describe surface energies and the adsorption energies of CO on metallic systems.[6,7] We set the cut-off energy for the plane waves to 400 eV. The first-order Methfessel–Paxton smearing technique[8] with a width of 0.2 eV was used, but the total energies obtained were extrapolated to zero width. Self-consistency was achieved when the total energy difference between the electronic optimization iterations was lower than 10^{-5} eV, and during geometry optimizations, a calculation converged when all forces were reduced to 2×10^{-2} eV Å^{-1}.

In this work, we consider that the adsorption of CO leads to the formation of CO* species while O_2 dissociates in contact with the surface forming two O* species. Here, * represents an adsorption site. These processes are represented by the following chemical equations:

$$\mathrm{CO} + * \rightarrow \mathrm{CO}^* \quad (1)$$

$$\mathrm{O_2} + ** \rightarrow 2\mathrm{O}^* \quad (2)$$

When equilibrated, these processes can be represented by equations 3 and 4, from which the typical Langmuir equations can be derived.

$$\frac{\theta_{\mathrm{CO}}}{1-\theta_{\mathrm{CO}}-\theta_{\mathrm{O}}} = p_{\mathrm{CO}}K_{\mathrm{CO}} = p_{\mathrm{CO}}\frac{1}{p^0}e^{-\Delta G_{\mathrm{CO}}(\theta_{\mathrm{CO}},\theta_{\mathrm{O}})/RT} \quad (3)$$

$$\frac{\theta_{\mathrm{O}}^2}{(1-\theta_{\mathrm{CO}}-\theta_{\mathrm{O}})^2} = p_{\mathrm{O_2}}K_{\mathrm{O_2}} = p_{\mathrm{O_2}}\frac{1}{p^0}e^{-\Delta G_{\mathrm{O}}(\theta_{\mathrm{CO}},\theta_{\mathrm{O}})/RT} \quad (4)$$

Here, p_{CO} and $p_{\mathrm{O_2}}$ are the partial pressures of CO and O_2, θ_{CO} and θ_{O} are the surface coverages of CO* and O* species, p^0 is the standard pressure or 1 bar, R is the ideal gas constant, and T is the temperature. The squared terms in equation 4 arise from the consideration of dissociative adsorption of O_2. Under the scheme described previously,[9,10] we accounted for lateral adsorbate–adsorbate interactions by introducing interaction parameters within the adsorption equilibrium constants, K_{CO} and $K_{\mathrm{O_2}}$. This modification is also known as the Fowler–Guggenheim isotherm[11] and accounts for changes in the adsorption energy due to surface coverage. In equations 3 and 4, $\Delta G_i(\theta_{\mathrm{CO}},\theta_{\mathrm{O}})$ is the coverage-dependent Gibbs free energy of adsorption of species i, estimated by considering no change in the zero-point energy correction due to the adsorption. Therefore, $\Delta G_i(\theta_{\mathrm{CO}},\theta_{\mathrm{O}}) = E_{\mathrm{ads},i} - T\Delta S_i$, where $\Delta S_i = S_{\mathrm{ads},i} - S_{\mathrm{gas},i}$ with the additional simplification that the entropy of the adsorbed system, $S_{\mathrm{ads},i}$, is zero. The interaction parameters w_{CO}, w_{O}, and $w_{\mathrm{CO,O}}$ modify the Gibbs free energy of adsorptions of CO* and O* according to equations 5 and 6:

$$\Delta G_{\mathrm{CO}}(\theta_{\mathrm{CO}},\theta_{\mathrm{O}}) = E_{\mathrm{CO}}(0,0) - z\left(w_{\mathrm{CO}}\theta_{\mathrm{CO}} + w_{\mathrm{CO,O}}\theta_{\mathrm{O}}\right) - T\Delta S_{\mathrm{CO}} \quad (5)$$

$$\Delta G_{\mathrm{O}}(\theta_{\mathrm{CO}},\theta_{\mathrm{O}}) = E_{\mathrm{O}}(0,0) - z\left(w_{\mathrm{O}}\theta_{\mathrm{O}} + w_{\mathrm{CO,O}}\theta_{\mathrm{CO}}\right) - T\Delta S_{\mathrm{O}} \quad (6)$$

The single-species interaction parameters, w_{CO} and w_{O}, are calculated as: $zw_i = E_i(0) - E_i(1)$. Here, z is the number of the nearest neighbors each adsorbate i would have on the surface under a high coverage situation, $\theta_i = 1$. Only the adsorption energies of species i at low $E_i(0)$ and high $E_i(1)$ coverages are required to estimate w_i. In practice, $E_i(0)$ is approximated by calculating the adsorption energy of species i in a sufficiently big slab. The details of the slab models considered here are described in the next section and shown in Supplementary Figures 23–27. The procedure to obtain $w_{\mathrm{CO,O}}$ is explained in detail elsewhere.[10] Here, we only describe the calculation of the interaction parameter $w_{\mathrm{CO,O}}$ for the $\{111\}$ facet, and a similar analysis can be used to derive appropriated equations for other surfaces.

In a high coverage situation where all sites of the $\{111\}$ surface are occupied by equal amounts of CO* and O*, each adsorbate has six nearest neighbors. In a slab model with dimensions of 2×2, the total energy change due to the adsorption of one O_2 (two O* species) and two CO molecules is represented by equation 7. $E_{\mathrm{ads(2\times2)}}$ is the total energy of the adsorbed system, $E_{\mathrm{slab(2\times2)}}$ is the energy of the clean surface slab, $E_{\mathrm{CO}}^{\mathrm{gas}}$ and $E_{\mathrm{O}}^{\mathrm{gas}}$ refer to the gas phase energy of CO and $\frac{1}{2}\mathrm{O}_2$. The individual contributions to the total adsorption energy for CO* and O* are represented by e_{CO} and e_{O}, respectively. If considering the adsorbed system presented in Supplementary Figure 23c, each CO* has four O* and two CO* nearest neighbors. Since e_{CO} represents the contribution of a single CO* to the adsorption energy in the mixed system at full coverage (Supplementary Figure 23c), the difference between $E_{\mathrm{CO}}(0)$ and e_{CO} energies is due to the adsorbate–adsorbate interactions, as given in equation 8. Two of these interactions are CO*–CO*, while four are CO*–O* (Supplementary Figure 23c). A similar equation 9 can be derived for the $E_{\mathrm{O}}(0)$ and e_{O} difference. By combining equations 7–9, one can solve for the interaction parameter $w_{\mathrm{CO,O}}$.

$$2e_{\mathrm{CO}} + 2e_{\mathrm{O}} = E_{\mathrm{ads(2\times2)}} - E_{\mathrm{slab(2\times2)}} - 2E_{\mathrm{CO}}^{\mathrm{gas}} - 2E_{\mathrm{O}}^{\mathrm{gas}} \quad (7)$$

$$E_{\mathrm{CO}}(0) - e_{\mathrm{CO}} = 2w_{\mathrm{CO}} + 4w_{\mathrm{CO,O}} \quad (8)$$

$$E_{\mathrm{O}}(0) - e_{\mathrm{O}} = 2w_{\mathrm{O}} + 4w_{\mathrm{CO,O}} \quad (9)$$

2. Surface models

For our calculations, we built {111}, {100}, {110}, {120}, and {311} surfaces and calculated low and high coverage situations for CO and O as adsorbates. For the low coverage situation, supercells of 3×3, 3×3, 2×2, 2×2, and 2×3 were used as surface models of the {111}, {100}, {110}, {120}, and {311} facets, respectively (Supplementary Figures 23–27, 35). We explored multiple configurations for the adsorbed systems and used only those with the lowest energy. In all the cases, all high coverage situations for a single adsorbate, $\theta_i = 1$, were simulated using 1×1 slabs. For the high coverage situations with both CO* and O* species on a surface, we used slab models with dimensions of 2×2, 2×2, 1×2, 1×1, and 1×2 for corresponding {111}, {100}, {110}, {120}, and {311} facets.

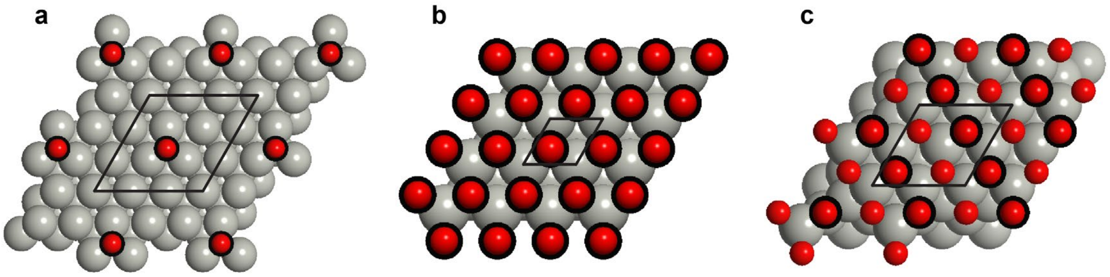

Supplementary Figure 23. Model of Pd {111} surface. (**a**) Low and (**b**) high coverage cases for CO adsorbed over the {111} surface of Pd. (**c**) High surface coverage case with a 1:1 mixture of CO and O adsorbed species used to calculate the interaction parameter $w_{\mathrm{CO,O}}$. Black, red, and grey spheres represent C, O, and Pd atoms, respectively. The limits of the unit cells are displayed with black lines.

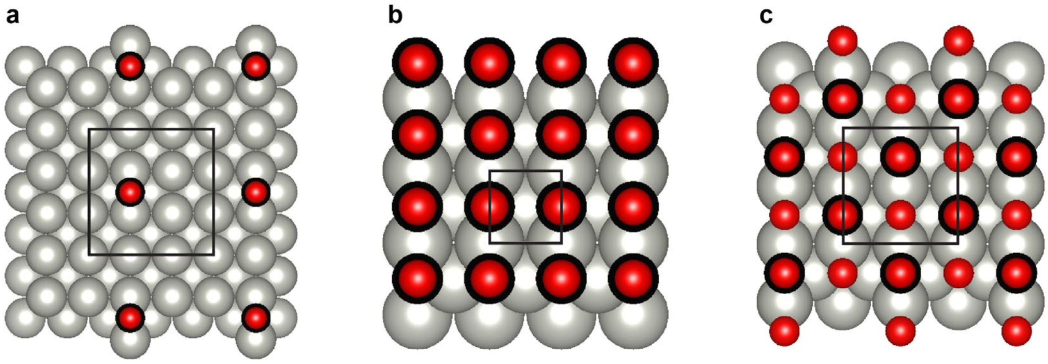

Supplementary Figure 24. Model of Pd {100} surface. (**a**) Low and (**b**) high coverage cases for CO adsorbed over the {100} surface of Pd. (**c**) High surface coverage case with a 1:1 mixture of CO and O adsorbed species used to calculate the interaction parameter $w_{\mathrm{CO,O}}$. Black, red, and grey spheres represent C, O, and Pd atoms, respectively. The limits of the unit cells are displayed with black lines.

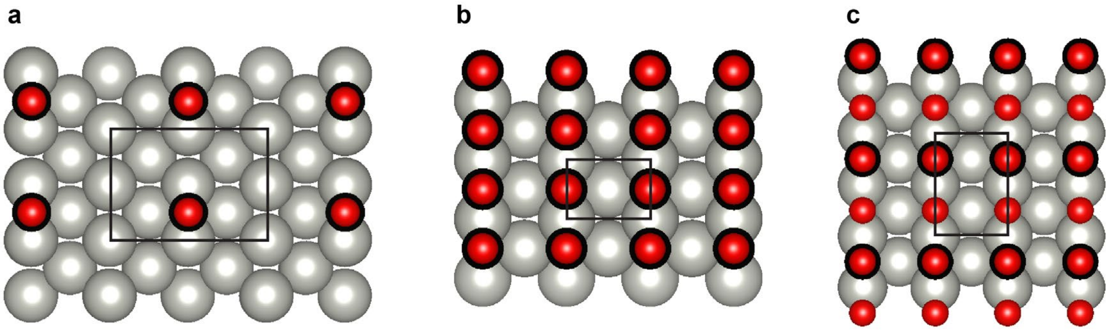

Supplementary Figure 25. Model of Pd {110} surface. (**a**) Low and (**b**) high coverage cases for CO adsorbed over the {110} surface of Pd. (**c**) High surface coverage case with a 1:1 mixture of CO and O adsorbed species used to calculate the interaction parameter $w_{\mathrm{CO,O}}$. Black, red, and grey spheres represent C, O, and Pd atoms, respectively. The limits of the unit cells are displayed with black lines.

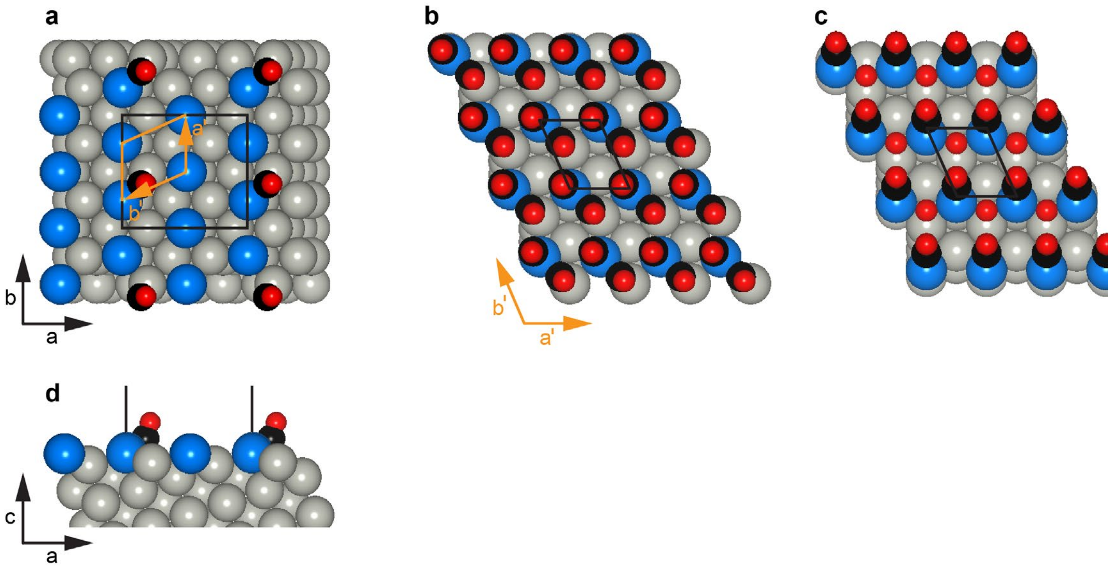

Supplementary Figure 26. Model of Pd {120} surface. (**a**) Low and (**b**) high coverage cases for CO adsorbed over the {120} surface of Pd. (**c**) High surface coverage case with a 1:1 mixture of CO and O adsorbed species used to calculate the interaction parameter $w_{CO,O}$. A conventional orthogonal supercell equivalent to 2×2 unit cells was used in (**a**), while a single unit cell was used in (**b**) and (**c**). The limits of the unit cells used for (**b**) and (**c**) are shown with yellow lines in (**a**). (**d**) Rotated view of (**a**) to better visualize Pd edge atoms (*blue spheres*). Black, red, and grey spheres represent C, O, and Pd atoms, respectively. The limits of the corresponding slab models are displayed with black lines.

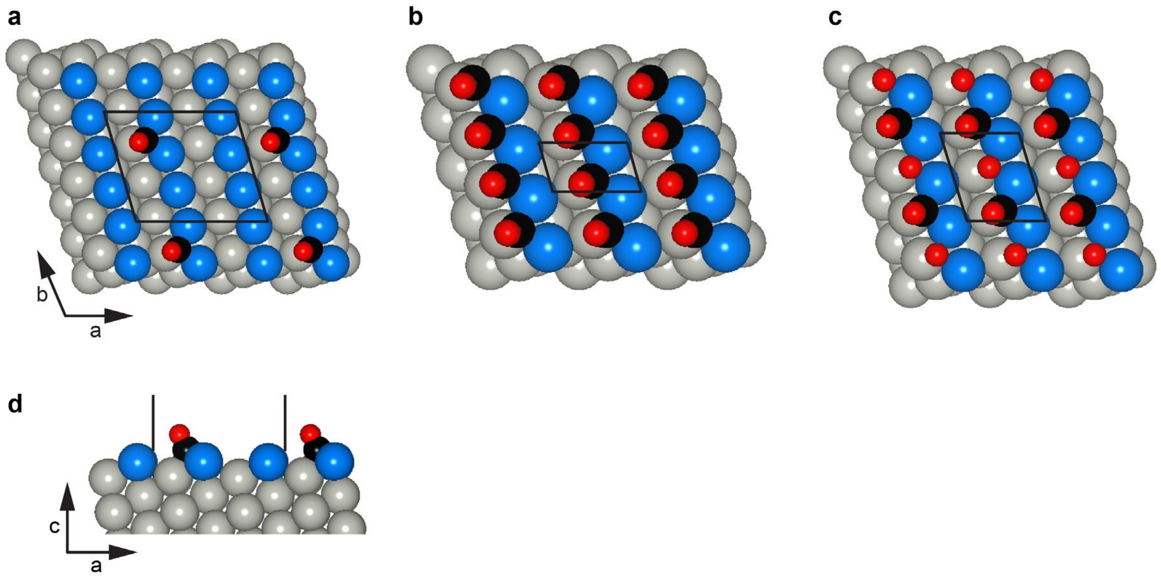

Supplementary Figure 27. Model of Pd {311} surface. (**a**) Low and (**b**) high coverage cases for CO adsorbed over the {311} surface of Pd. (**c**) High surface coverage case with a 1:1 mixture of CO and O adsorbed species used to calculate the interaction parameter $w_{CO,O}$. (**d**) Rotated view of (**a**) to better visualize Pd edge atoms colored in blue. Black, red, and grey spheres represent C, O, and Pd atoms, respectively. The limits of the unit cells are displayed with black lines.

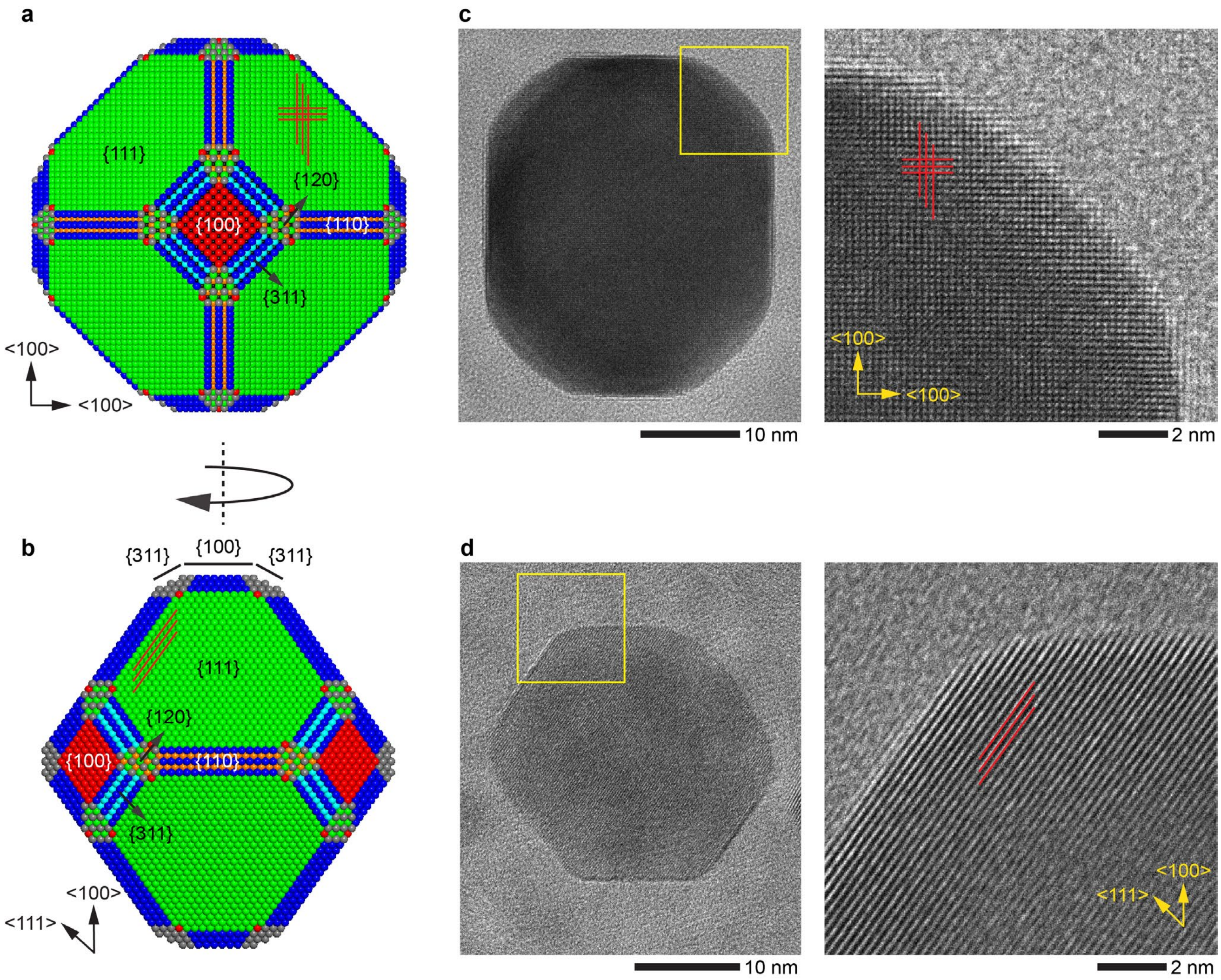

Supplementary Figure 28. Model and TEM images of Pd NPs. (**a**–**b**) Two views of a constructed Pd NP displaying its various exposed facets. These views differ by a 45° rotation angle. Low- and high-resolution TEM images of the corner areas of (**c**) the truncated nanocube and (**d**) nano-octahedron shown in Figures 2 and 3. The constructed NP shown in (**a**) is consistent with the experimentally observed truncated nanocube in (**c**), while the constructed NP shown in (**b**) agrees with nano-octahedron in (**d**). Remarkably, the patterns of atomic columns indicated by red lines in (**c**) and (**d**) nicely match the patterns in the respective modeled NPs shown in (**a**) and (**b**).

3. Effects of ${p_{\mathrm{CO}}}/{p_{\mathrm{O_2}}}$ ratio and temperature on the morphology of Pd NP

To assess the effects of the reaction conditions, the ${p_{\mathrm{CO}}}/{p_{\mathrm{O_2}}}$ ratio and the temperature, on the morphology of the Pd NPs, we determine the changes in the interface tension energies, $\gamma_{hkl}^{\mathrm{int}}$, and the number of surface atoms with these parameters. Because only the ratio of $\gamma_{hkl}^{\mathrm{int}}$ between the facets is important for the construction of NPs following Wulff's scheme, we calculated the ratios of interface tension energies of each facet, with respect to that of the $\{111\}$ facet, $\gamma_{111}^{\mathrm{int}}$. Supplementary Figure 29a shows the ratio $\gamma_{hkl}^{\mathrm{int}}/\gamma_{111}^{\mathrm{int}}$ as a function of temperature. It is evident that the ratio $\gamma_{hkl}^{\mathrm{int}}/\gamma_{111}^{\mathrm{int}}$ shifts to higher temperatures with higher ${p_{\mathrm{CO}}}/{p_{\mathrm{O_2}}}$ ratios. The crossing points between $\gamma_{100}^{\mathrm{int}}/\gamma_{111}^{\mathrm{int}}$, $\gamma_{110}^{\mathrm{int}}/\gamma_{111}^{\mathrm{int}}$, $\gamma_{120}^{\mathrm{int}}/\gamma_{111}^{\mathrm{int}}$, and $\gamma_{311}^{\mathrm{int}}/\gamma_{111}^{\mathrm{int}}$ curves shift from 307 °C at a ${p_{\mathrm{CO}}}/{p_{\mathrm{O_2}}}$ ratio of 0.5, to 377 °C at a ${p_{\mathrm{CO}}}/{p_{\mathrm{O_2}}}$ ratio of 2.0. From these results, we can expect that changes in the shape of NPs occur at higher temperatures under atmospheres richer in CO. In fact, the experimentally measured ignition temperature shifts to higher values at higher CO content (Figure 4d), in good agreement with the theoretical calculations obtained here. We can understand the changes in $\gamma_{hkl}^{\mathrm{int}}/\gamma_{111}^{\mathrm{int}}$ at various ${p_{\mathrm{CO}}}/{p_{\mathrm{O_2}}}$ ratios (Supplementary Figure 29a) by looking into the dependency of CO coverage (θ_{CO}) in Supplementary Figure 32, and their slopes ($\mathrm{d}\theta_{\mathrm{CO}}/\mathrm{d}T$) in Supplementary Figure 33. The lower the ${p_{\mathrm{CO}}}/{p_{\mathrm{O_2}}}$ ratio, the faster θ_{CO} decreases with temperature (Supplementary Figures 32–33). Because $\gamma_{hkl}^{\mathrm{int}}$ is tightly linked to θ_{CO}, the differences observed in Supplementary Figure 29a are a consequence of the changes in the rate at which CO adsorbs and desorbs over the various facets.

At first glance, the shape of the curves presented in Supplementary Figure 29a changes at various ${p_{\mathrm{CO}}}/{p_{\mathrm{O_2}}}$ ratios. To study in detail these apparent differences, we analyzed the change in the ratios $\gamma_{hkl}^{\mathrm{int}}/\gamma_{111}^{\mathrm{int}}$ with respect to the temperature, ${\Delta(\gamma_{hkl}^{\mathrm{int}}/\gamma_{111}^{\mathrm{int}})}/{\Delta T}$, *i.e.*, the slope of $\gamma_{hkl}^{\mathrm{int}}/\gamma_{111}^{\mathrm{int}}$ curves as a function of temperature (Supplementary Figure 29b). At lower ${p_{\mathrm{CO}}}/{p_{\mathrm{O_2}}}$ ratios, changes in $\gamma_{hkl}^{\mathrm{int}}/\gamma_{111}^{\mathrm{int}}$ occur faster, as evidenced by sharper and more prominent peaks in Supplementary Figure 29b. Therefore, not only the changes in the shape of the NPs are expected to occur at a lower temperature for lower ${p_{\mathrm{CO}}}/{p_{\mathrm{O_2}}}$, but these changes are expected to occur in a smaller temperature range.

To verify how these effects, *i.e.*, temperature and ${p_{\mathrm{CO}}}/{p_{\mathrm{O_2}}}$ ratio, translate to actual morphological changes in the NPs, we built models of Pd NPs comprising about 60000 atoms with diameters of 13–15 nm, under various ${p_{\mathrm{CO}}}/{p_{\mathrm{O_2}}}$ ratios. We explored the relevant temperature range, in steps of 5 °C, where the NPs undergo the most prominent changes in shape (Figure 5a and Supplementary Table 1). We assessed the morphological changes in the NPs with changes in the temperature by counting the number of surface atoms with specific coordination numbers (CN), as shown in Figure 5a. To obtain a better perspective about the NP morphology, the CNs have been translated to the associated facets, which are listed in Supplementary Table 2. At a ${p_{\mathrm{CO}}}/{p_{\mathrm{O_2}}}$ ratio of 1.0,

atoms with CN of 7 and 11 (from {110} facets) dominate the surface at temperatures lower than 290 °C. Above this temperature, the NP experience a structural rearrangement as evidenced by the sudden decrease of surface atoms with CN of 7 and 11 and a simultaneous increase in the surface atoms with CN of 9 (from {111} facets). Few snapshots of the modeled NPs before, during, and after the morphological changes are shown in Supplementary Figure 30. To measure how fast the NPs change in shape, on the scale of temperature, we determine the temperature span (δT_{span}) at which the NPs undergo the major morphological transformations, as shown in Supplementary Figure 30a.

According to our calculations, the temperature span in which the NPs experience major structural changes increases with increasing $p_{\mathrm{CO}}/p_{\mathrm{O_2}}$ ratio (Supplementary Table 1 and Supplementary Figure 31). This indicates that at larger $p_{\mathrm{CO}}/p_{\mathrm{O_2}}$ ratios, greater changes in temperature are required to produce changes in the shape of the NPs. In our experiments, we have seen a sudden increase in the reaction temperature occurs right at the switch from low- to high-activity states. This sudden increase in temperature is indicated as δT in Figure 4a. Our experimental results show that this δT parameter increases with increasing $p_{\mathrm{CO}}/p_{\mathrm{O_2}}$ ratio (Figure 4f). Specifically, δT increases from 1.5 °C at $p_{\mathrm{CO}}/p_{\mathrm{O_2}} = 0.5$ to 11 °C at $p_{\mathrm{CO}}/p_{\mathrm{O_2}} = 2.0$. Therefore, we propose that the observed increase in δT with increasing $p_{\mathrm{CO}}/p_{\mathrm{O_2}}$ ratio is a consequence of the larger temperature changes required to produce the same changes in the NP shape, as shown by our modeling results.

Supplementary Table 1. Range of temperatures at which the NPs at a given $p_{\mathrm{CO}}/p_{\mathrm{O_2}}$ ratio experience major structural transformations, and the temperature span (δT_{span}) required to produce this structural change.

$p_{\mathrm{CO}}/p_{\mathrm{O_2}}$	Temperature range (°C)	δT_{span} (°C)
0.5	265–305	40
1.0	290–335	45
1.5	295–350	55
2.0	305–365	60

Supplementary Table 2. Coordination numbers (CN) of surface atoms belonging to specific facets.

Facet	CN
{111}	9
{100}	8
{110}	7, 11
{120}	6, 9, 11
{311}	7, 10

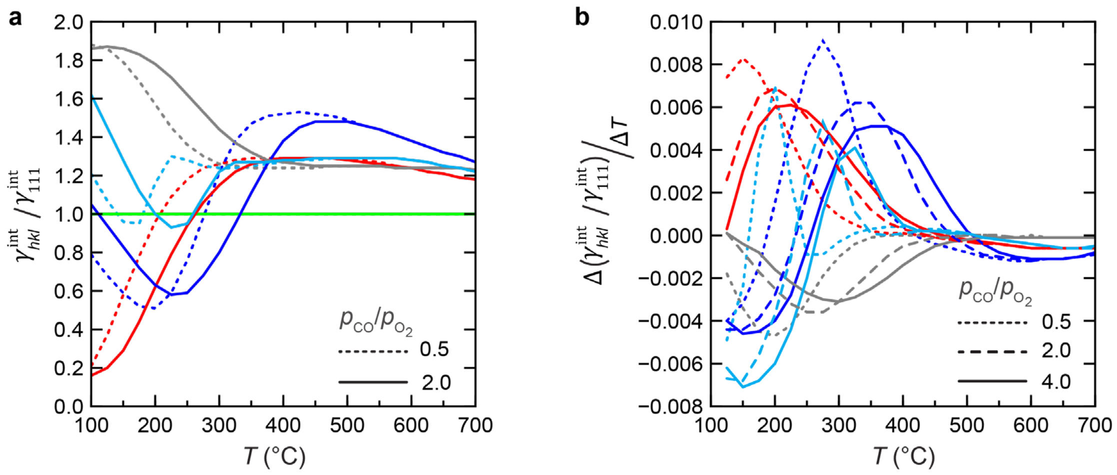

Supplementary Figure 29. Interface tension energies of different Pd facets under reaction conditions. (**a**) Ratios of interface tension energies of {100}, {110}, {120}, and {311} facets to that of the {111} facet as a function of temperature at $p_{\mathrm{CO}}/p_{\mathrm{O_2}}$ = 0.5 and 2.0. (**b**) Rate of change of the interface tension energy ratios with respect to temperature at three different $p_{\mathrm{CO}}/p_{\mathrm{O_2}}$ ratios. Different colors correspond to different $\{hkl\}$ facets: green – {111}, red – {100}, blue – {110}, grey – {120}, and cyan – {311}.

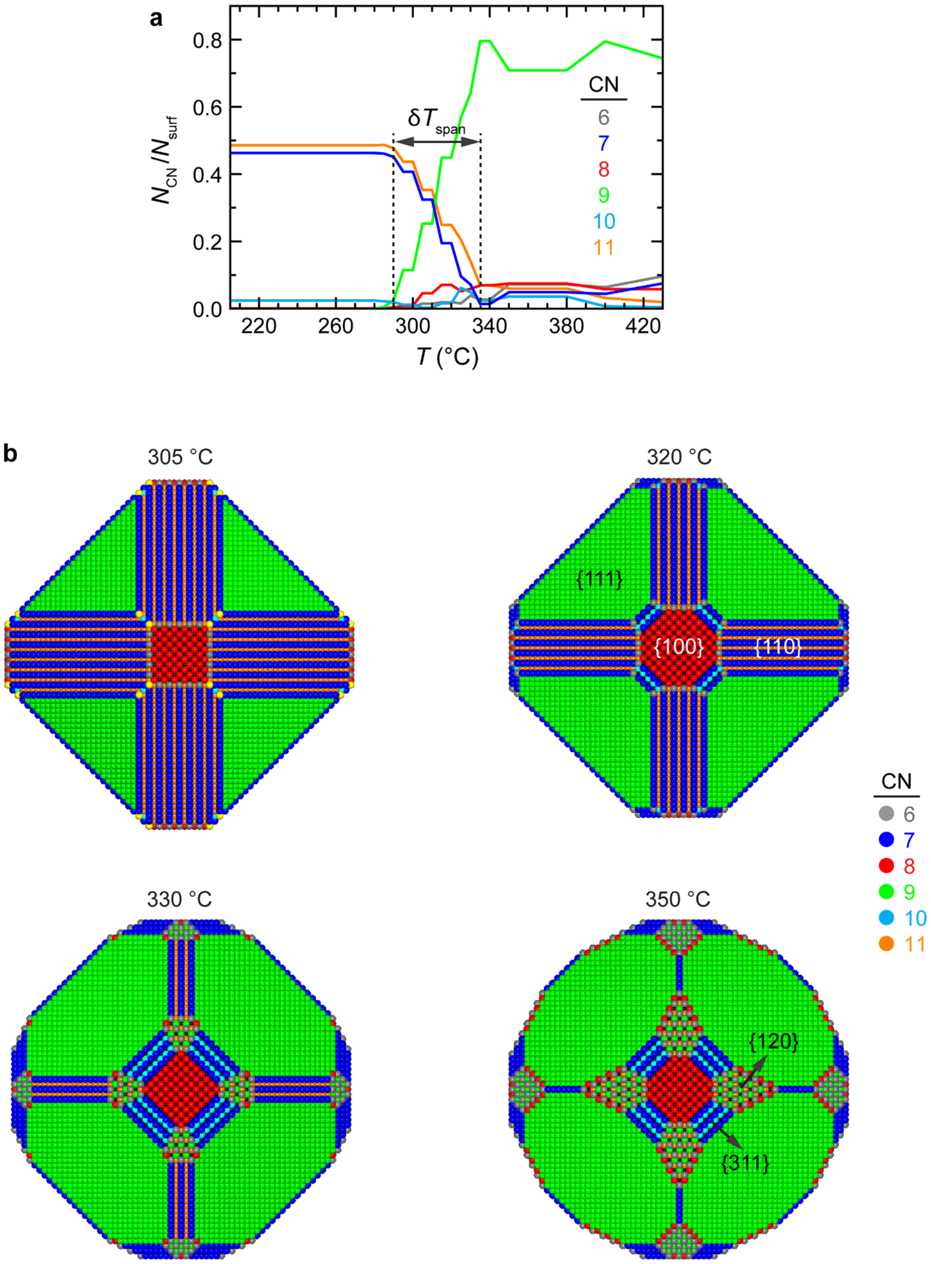

Supplementary Figure 30. Models of Pd NPs under reaction conditions. (**a**) Fraction of the surface atoms with different coordination numbers (CNs) in Pd NPs, $^{N_{CN}}/_{N_{surf}}$, generated at $^{p_{CO}}/_{p_{O_2}} = 1.0$, with O_2 partial pressure (p_{O_2}) of 140 Torr. N_{surf} is the total number of surface atoms. Each colored curve corresponds to the fraction of surface atoms with a CN indicated by color labels. (**b**) Snapshots of the NP at 305, 320, 330, and 350 °C. The atoms were colored according to their CNs. The modeled NPs have around 60000 atoms with diameters between 13–15 nm. The yellow color atoms in (**b**) correspond to CN of 5, which is not shown in the plots in (**a**).

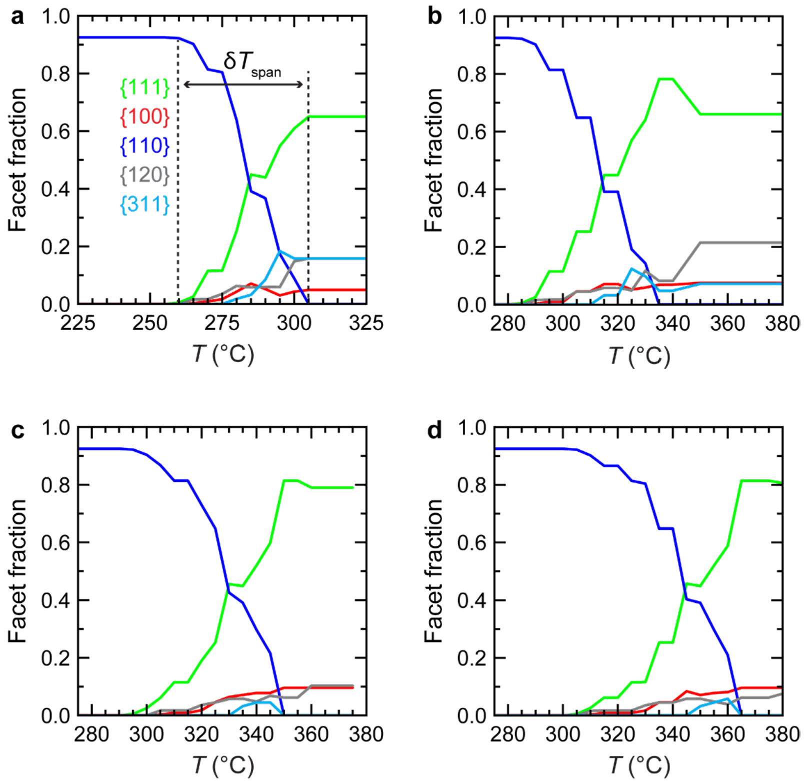

Supplementary Figure 31. The surface structure of Pd NP under different reaction conditions. Estimated fraction of the number of surface atoms from each facet of Pd NPs at $^{p_{\mathrm{CO}}}/_{p_{\mathrm{O_2}}}$ ratios of (**a**) 0.5, (**b**) 1.0, (**c**) 1.5, and (**d**) 2.0, based on the coordination number (CN) of surface atoms. The partial pressure of O_2 was fixed to 140 Torr. The fraction of the surface atoms in {111} and {100} can be directly estimated with the surface atoms with CNs of 9 and 8, respectively. Because {110}, {120}, and {311} facets expose atoms with more than one distinct CN, we estimated their contributions by counting the number of the surface atoms with a unique CN and then multiplied it by the number of distinct atoms in that facet. For example, for the facet {311}, which exposes atoms with CNs of 7 and 10, we estimated the number of surface atoms from {311} by multiplying this number of surface atoms with the CN of 10 by 2. Only facet {311} exposes atoms with the CN of 10.

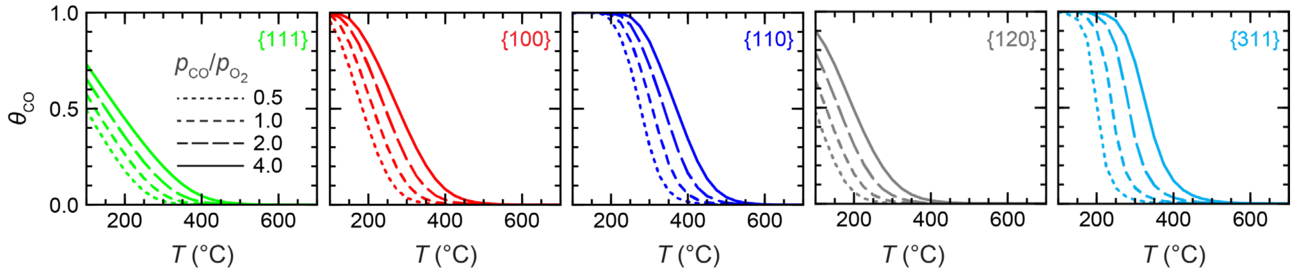

Supplementary Figure 32. CO coverage of different Pd facets under reaction conditions. Temperature dependence of CO coverage (θ_{CO}) over the {111}, {100}, {110}, {120}, and {311} Pd facets at different p_{CO}/p_{O_2} ratios as indicated in the inset of the first panel.

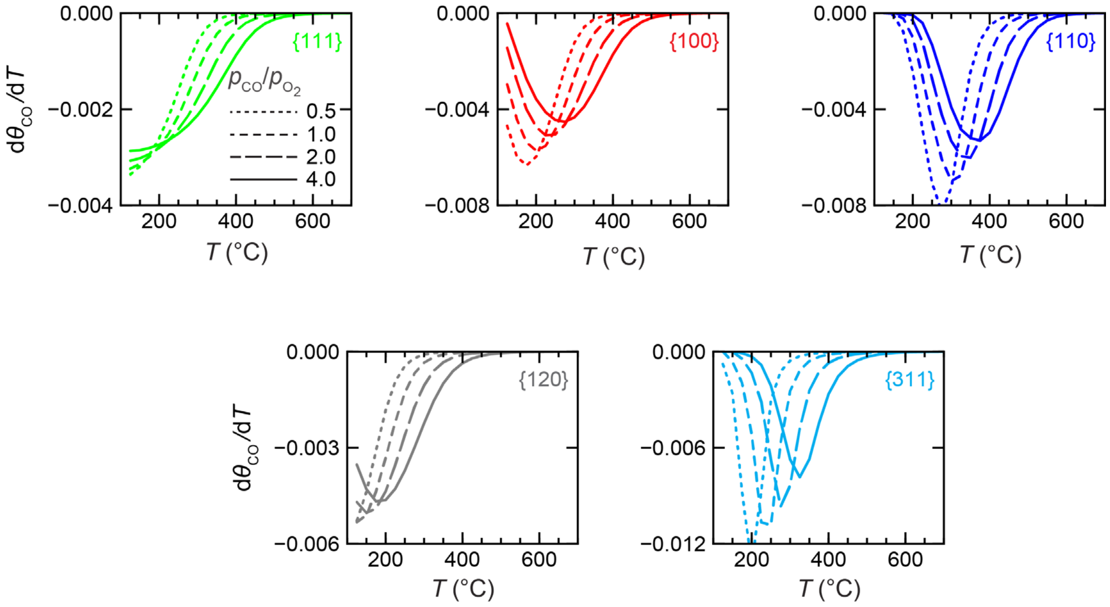

Supplementary Figure 33. Rates of change of CO coverage for different Pd facets under reaction conditions. Temperature dependence of the rate of change of CO coverage ($d\theta_{CO}/dT$) over the {111}, {100}, {110}, {120}, and {311} Pd facets at different p_{CO}/p_{O_2} ratios as indicated in the inset of the first panel.

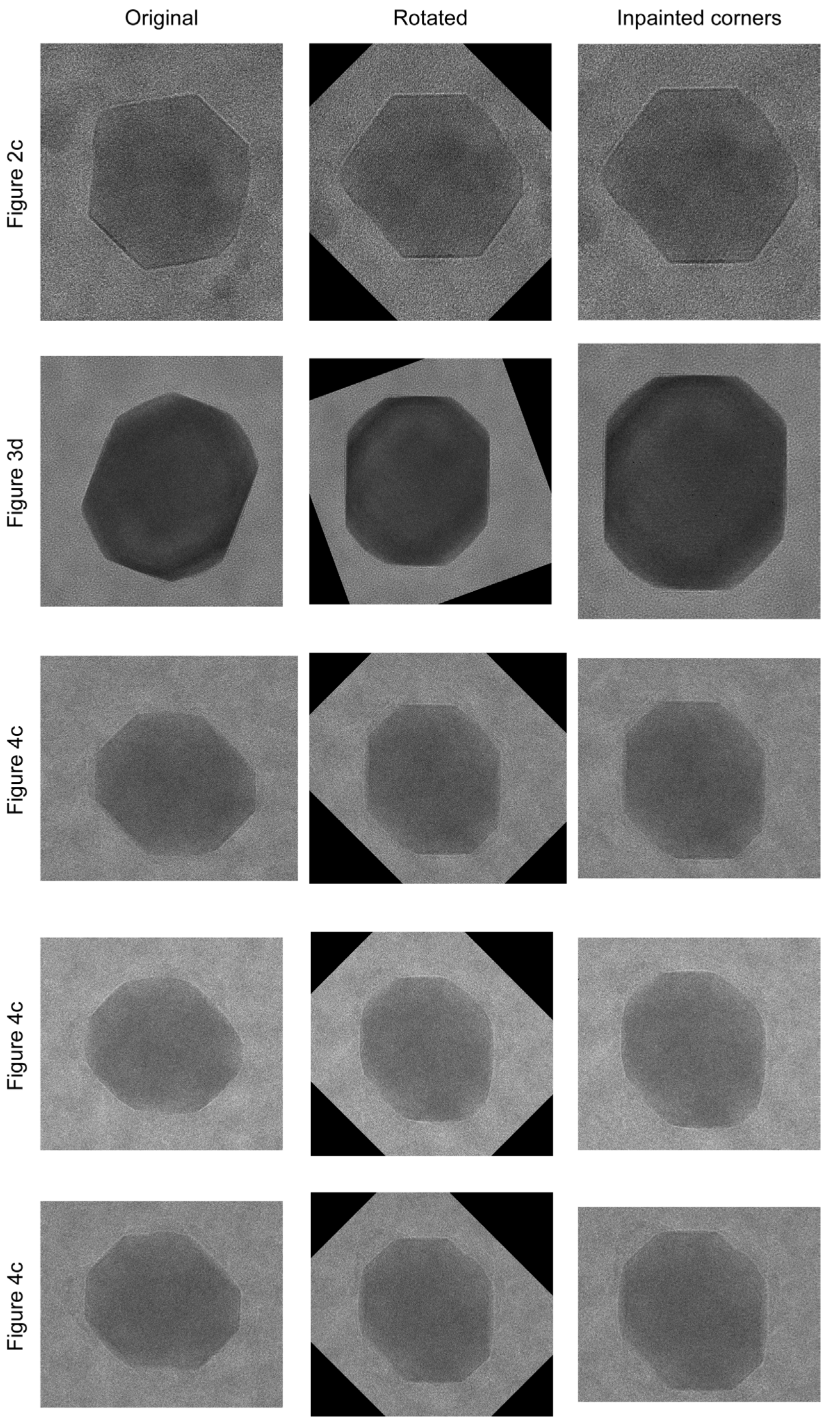

Supplementary Figure 34. Processing of the images displayed in the text. Original, rotated, and false-filled images used in Figures 2c, 3d, and 4c.

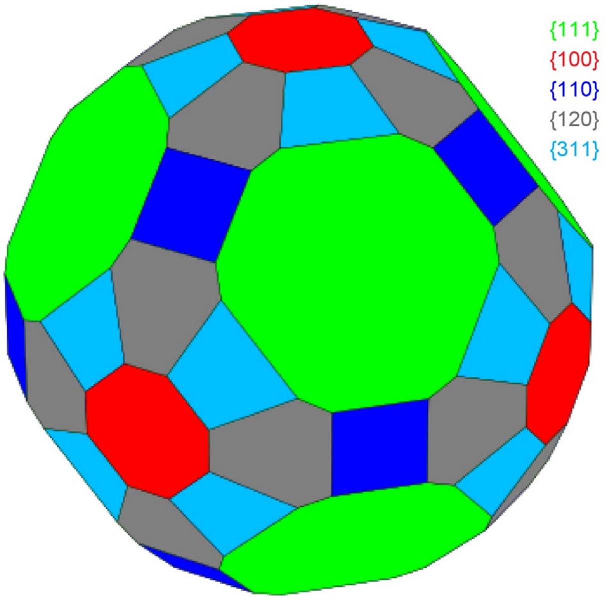

Supplementary Figure 35. Surface of Pd NP. Schematic showing different facets of a Pd NP.

Supplementary references

1 Kresse, G. & Hafner, J. Ab initio molecular dynamics for liquid metals. *Phys. Rev. B* **47**, 558–561 (1993).

2 Kresse, G. & Hafner, J. Ab initio molecular-dynamics simulation of the liquid-metal–amorphous-semiconductor transition in germanium. *Phys. Rev. B* **49**, 14251–14269 (1994).

3 Kresse, G. & Furthmüller, J. Efficient iterative schemes for ab initio total-energy calculations using a plane-wave basis set. *Phys. Rev. B* **54**, 11169–11186 (1996).

4 Kresse, G. & Furthmüller, J. Efficiency of ab-initio total energy calculations for metals and semiconductors using a plane-wave basis set. *Comput. Mater. Sci.* **6**, 15–50 (1996).

5 Kresse, G. & Joubert, D. From ultrasoft pseudopotentials to the projector augmented-wave method. *Phys. Rev. B* **59**, 1758–1775 (1999).

6 Wellendorff, J. *et al.* Density functionals for surface science: Exchange-correlation model development with Bayesian error estimation. *Phys. Rev. B* **85**, 235149 (2012).

7 Gautier, S., Steinmann, S. N., Michel, C., Fleurat-Lessard, P. & Sautet, P. Molecular adsorption at Pt(111). How accurate are DFT functionals? *Phys. Chem. Chem. Phys.* **17**, 28921–28930 (2015).

8 Methfessel, M. & Paxton, A. T. High-precision sampling for Brillouin-zone integration in metals. *Phys. Rev. B* **40**, 3616–3621 (1989).

9 Zhu, B., Meng, J. & Gao, Y. Equilibrium shape of metal nanoparticles under reactive gas conditions. *J. Phys. Chem. C* **121**, 5629–5634 (2017).

10 Meng, J., Zhu, B. & Gao, Y. Shape evolution of metal nanoparticles in binary gas environment. *J. Phys. Chem. C* **122**, 6144-6150 (2018).

11 Fowler, R. H. & Guggenheim, E. A. *Statistical Thermodynamics*, Ch. 10 (Cambridge University Press, Cambridge, 1939).
