# Supplementary material for: Periodic structural changes in Pd nanoparticles during oscillatory CO oxidation reaction
Source: Nat Commun. 2022 Oct 19;13:6176. doi: 10.1038/s41467-022-33304-x (PMC9582216; doi:10.1038/s41467-022-33304-x)
Supplement: Supplementary file 3 — Description of Additional Supplementary Files [file 41467_2022_33304_MOESM3_ESM.pdf]

Description of Additional Supplementary Files

File Name: Supplementary Movie 1
Description: Movie describing the structural changes in the Pd nano-octahedron shown in Figure 2 during an oscillatory reaction at a temperature of 380 °C and a gas pressure ratio of $p_{CO}/p_{O_2} \approx 0.5$.

File Name: Supplementary Movie 2
Description: Movie describing the structural changes in the truncated Pd nanocube shown in Figure 3 during an oscillatory reaction at a temperature of 420 °C and a gas pressure ratio of $p_{CO}/p_{O_2} \approx 0.5$.

File Name: Supplementary Movie 3
Description: Movie describing the structural changes in the truncated Pd nanocube shown in Figure 4 during an oscillatory reaction at a temperature of 460 °C and a gas pressure ratio of $p_{CO}/p_{O_2} \approx 0.8$.

File Name: Supplementary Movie 4
Description: Movie describing the structural changes in the truncated Pd nanocube shown in Figure 4 during a reaction in a temperature range of 460 to 540 °C and a gas pressure ratio of $p_{CO}/p_{O_2} \approx 2.0$.
